# Co-designing a post-discharge intervention to improve mobility recovery among patients with lower limb fractures using human-centered design in greater Kampala, Uganda

**DOI:** 10.1371/journal.pone.0360232

**Published:** 2026-11-10

**Authors:** Swaibu Zziwa, Erisa Sabakaki Mwaka, Nukhba Zia, Esther Buregyeya, Olive Kobusingye, Fred Nuwaha

**Affiliations:** 1 Department of Disease Control and Environmental Health, Makerere University School of Public Health, Kampala, Uganda; 2 Department of Public Health, Faculty of Health Sciences, Mountains of the Moon University, Fort Portal, Uganda; 3 Department of Anatomy, School of Biomedical Sciences, College of Health Sciences, Makerere University, Kampala, Uganda; 4 Department of International Health, Health Systems Program, Johns Hopkins Bloomberg School of Public Health, Baltimore, Maryland, United States of America; 5 ClinRé Foundation, Arlington, Virginia, United States of America; Shuguang Hospital Affiliated to Shanghai University of Traditional Chinese Medicine, CHINA

## Abstract

**Introduction:**

Lower limb fractures are a major cause of functional limitation and require coordinated post-discharge care to support recovery and prevent complications. In low- and middle-income countries, including Uganda, continuity of care after discharge is often limited by health system constraints, socioeconomic barriers, and community barriers. This study aimed to co-design a post-discharge intervention to improve recovery among patients with lower limb fractures in Greater Kampala, Uganda, using a Human-Centered Design (HCD) approach.

**Methods:**

A qualitative exploratory study guided by the HCD participatory and iterative framework was conducted between July and December 2025. Stakeholders including healthcare providers, community health workers, patients, and caregivers participated in focus group discussions, key informant interviews, and in-depth interviews to explore their experiences, needs, and challenges related to post-discharge care. Stakeholders subsequently participated in an iterative co-design process to prioritize and refine intervention components. Data were audio-recorded, transcribed verbatim, and analyzed thematically using NVivo version 15.

**Results:**

Several interconnected barriers to post-discharge care were identified, including discontinuity of care after hospital discharge, inadequate discharge information, transportation challenges, community and family influences on treatment decisions, use of traditional bone setters, and health-system limitations. Through the co-design process, stakeholders proposed intervention strategies comprising structured discharge preparation, improved documentation of patient and caregiver contact information, strengthened linkages between health facilities and community health workers, community-based follow-up, and decentralized follow-up services at lower-level health facilities.

**Conclusions:**

Health-system and community-level factors influenced post-discharge care among patients with lower limb fractures. The study co-designed an integrated facility–community intervention comprising structured discharge processes, strengthened community health worker linkages, and community-based follow-up. Further studies should evaluate the feasibility and effectiveness of the intervention in improving post-discharge care and recovery outcomes.

## Introduction

Globally, bone fractures are a leading cause of functional limitation and contribute substantially to reduced quality of life [1]. According to the Global Burden of Disease (GBD) 2021, an estimated 172.8 million new fracture cases and 453.3 million prevalent cases were reported globally [2,3]. In the same year, bone fractures contributed to approximately 25.2 million years lived with disability (YLDs), highlighting the substantial impact of these injuries on population health [2,3].

Lower limb fractures constitute a significant proportion of these injuries. Epidemiological analyses indicate that fractures involving the patella, tibia, fibula, and ankle are among the most common fracture types worldwide, with an age-standardized incidence rate of approximately 419.9 cases per 100,000 population in 2019 [1,4]. These fractures accounted for about 15.5 million years lived with disability globally, demonstrating their considerable contribution to the overall burden of musculoskeletal injury [1,4,5].

In Uganda, bone fractures are increasingly recognized as a major public health concern [6]. Previous studies conducted in Uganda have demonstrated an increasing burden of bone fractures, particularly lower-limb fractures, which are commonly associated with road traffic injuries and other traumatic events [6–10]. Lower-limb fractures, including those involving the femur, tibia, fibula, and other lower extremities, accounted for 68.4% of all traumatic injuries reported at Mulago National Referral Hospital [7].

Studies have further highlighted that delayed hospital presentation and limited access to post discharge care contributed to poor fracture recovery outcomes [6,10,11]. Although referral hospitals provide specialized orthopedic care, post-discharge rehabilitation services remain limited, and continuity of care between hospitals and communities is often weak. Many patients return home after discharge without structured follow-up systems to monitor recovery or identify complications early [12]. Therefore, there is a need to design context-specific interventions that can strengthen the transition from the hospital to the community.

Post-discharge care plays a critical role in ensuring proper healing, preventing complications, and restoring functional mobility after a bone fracture [13]. Evidence suggests that coordinated care processes and structured rehabilitation programs significantly improve recovery outcomes for patients with bone fractures [14–18]. Post-discharge interventions such as physiotherapy, home-based exercise programs, and patient education have been shown to enhance functional recovery and reduce long-term functional limitation [14,19–22].

Effective post-discharge care is therefore an essential component of orthopedic care. Integrating community health workers into the follow-up care, improving discharge counseling, and developing structured monitoring systems could help bridge the gap between hospital treatment and community recovery [23,24]. However, designing effective interventions requires a deep understanding of patient experiences, health system constraints, and community dynamics [25].

Human-Centered Design (HCD) offers a promising approach to addressing these challenges. The HCD is a participatory design methodology that places end users at the center of intervention development, emphasizing empathy, collaboration, and iterative problem-solving [26–29]. By engaging healthcare providers, community health workers, and other stakeholders in the design process, HCD can generate contextually appropriate solutions that are more likely to be accepted and sustained within health systems [25,30–32]. Participatory design approaches have increasingly been applied in global health to develop innovative interventions that respond to local needs and realities [25].

However, despite the increasing application of HCD in health intervention development, there is limited evidence on its use to design post-discharge care and mobility recovery interventions among patients with lower-limb fractures specifically in Uganda. Previous studies have primarily focused on the burden and clinical management of fractures [6,7,11], while limited attention has been given to the collaborative development of interventions that address the challenges patients experience after leaving the hospital. In particular, little is known about how patients, caregivers, healthcare providers, and community stakeholders can work together to develop feasible and contextually appropriate solutions to strengthen the transition from hospital to community-based care.

The HCD is suitable for developing a post-discharge intervention, particularly for patients with lower-limb fractures, because recovery extends beyond hospital-settings and is influenced by patients’ experiences, caregiver support, physical environments, access to rehabilitation services, and interactions with healthcare and community systems. These factors are context-specific and may not be adequately addressed through conventional intervention development approaches such as theory-based approaches [33], which may not sufficiently capture the lived experiences, needs, barriers, preferences, and decision-making of end users.

In Uganda, research has been limited on how health system and community stakeholders can collaboratively design solutions to improve post-discharge care for patients with lower limb bone fractures. To the best of the authors’ knowledge, this is the first study in Uganda to apply a HCD approach to co-design a post-discharge intervention to improve mobility recovery among patients with lower-limb fractures. Unlike previous studies that have predominantly focused on the burden and clinical management of fractures [7,11,34], this study brings together the perspectives and experiences of patients, caregivers, healthcare providers, and community stakeholders to identify post-discharge needs, barriers, and feasible solutions. Therefore, this study aimed to co-design a post-discharge intervention to improve mobility recovery among patients with lower limb fractures in Greater Kampala using a HCD approach.

## Methods

**Study design:** A qualitative exploratory study guided by the HCD participatory and iterative framework was conducted to co-design a post-discharge intervention aimed at improving mobility recovery among patients with lower limb fractures. This design actively engaged key stakeholders involved in the management, rehabilitation, and recovery of patients with lower limb fractures**,** including patients, caregivers, healthcare providers, and community health workers, throughout the intervention co-design process. The HCD process involved exploring stakeholders’ experiences of post-discharge care, identifying barriers and gaps affecting mobility recovery, generating potential solutions, and collaboratively refining and prioritizing intervention components based on their relevance, acceptability, feasibility, and sustainability within the local health system and community context.

**Study setting:** The study was conducted in Greater Kampala Metropolitan Area (GKMA), which comprises six districts, including Kampala, Wakiso, Mukono, Buikwe, Mpigi, and Luweero [35]. This area is the largest urban and peri-urban hub in Uganda and hosts several national and regional referral hospitals, private health facilities, and specialized orthopedic centers that manage a high volume of trauma and fracture cases.

Study participants: The study included 94 participants, comprising 30 healthcare providers**,** including orthopedic surgeons, orthopedic officers, physiotherapists, occupational therapists, clinical officers, and nurses; 34 community health workers; 15 patients with lower limb bone fractures; and 15 caregivers. Participants were recruited between July and December 2025**.** Written informed consent was obtained from all participants prior to their enrolment in the study.

**Sampling procedure:** The study employed a purposive sampling technique to recruit study participants. Healthcare providers and community health workers were purposively selected from health facilities in Greater Kampala, including Mulago National Referral Hospital, Mukono General Hospital, Kawolo General Hospital, Kiwoko Hospital, and Mpigi Health Centre IV. Healthcare providers were eligible to participate in the study if they were directly involved in the clinical management, rehabilitation, discharge planning, or follow-up of patients with lower limb fractures, and included orthopedic surgeons, orthopedic officers, physiotherapists, occupational therapists, nurses, and clinical officers whereas. Community health workers were eligible if they were involved in community-based care, follow-up, referral, or linkage of patients to hospital services.

Patients and caregivers were also purposively selected from communities within Greater Kampala**.** The research assistants reviewed the medical records from the orthopedic department at Mulago National Referral Hospital to identify eligible patients and caregivers to participate in the study. Contact information obtained from the records was used to make phone calls and schedule appointments with eligible participants. Patients and caregivers who met the eligibility criteria were listed and visited either at their homes or at nearby health facilities. Patients were eligible if they had sustained a lower limb fracture, had been discharged from Mulago National Referral Hospital in the last one year, and were able to provide informed consent, whereas caregivers were eligible if they were directly involved in providing care or support to patients with lower limb bone fractures. Patients and caregivers who were unable to provide informed consent, or were unable to participate in the study for some reasons were excluded from the study (S1 Fig).

**Data Collection:** Data were collected through eight focus group discussions (FGDs) conducted with key stakeholders involved in the care and follow-up of patients with lower limb fractures. Of these, four FGDs were conducted with healthcare providers (including orthopedic surgeons, physiotherapists, nurses, and clinical officers) and four with community health workers, particularly members of VHTs. Prior to data collection, the researchers introduced themselves, and participants were informed about the study, its objectives, the data collection procedures, and the anticipated benefits of the study findings. Participants were also allowed to ask questions and seek clarification about the study before providing informed consent and participating in the discussions. Each discussion comprised 5–7 participants and lasted approximately 60–90 minutes**.**

The FGDs were facilitated using a semi-structured discussion guide developed by the research team and rehabilitation experts to encourage open dialogue and participation. The discussion guides were pretested with ten [10] participants with similar characteristics prior to data collection**.** Feedback from the pretesting was used to refine the wording, improve clarity, and sequencing of the questions before the guides were used in the study. Discussions explored participants’ experiences with post-discharge fracture care, perceived barriers to follow-up and rehabilitation services, community and socio-cultural influences on seeking rehabilitation care decisions, and potential strategies that could improve patient recovery and continuity of care after discharge. Data collection continued iteratively until data saturation was reached after four FGDs with healthcare providers and four FGDs with community health workers as subsequent discussions did not generate substantially new themes or intervention needs.

In addition, five key informant interviews (KIIs) were conducted with orthopedic surgeons**,** 15 in-depth interviews (IDIs) were conducted with patients with lower-limb bone fractures, and 15 IDIs with caregivers of patients with bone fractures**.** The guides were pretested with 13 participants**,** comprising of three healthcare providers, five patients, and five caregivers prior to data collection. Feedback from the pretesting was used to improve clarity, and sequencing of questions before the actual data collection. The interviews explored the lived experiences of patients and caregivers during the recovery process following hospital discharge. The KIIs and IDIs further explored challenges related to mobility recovery, access to rehabilitation services, adherence to recommended care, caregiver support, and barriers encountered during home-based care. Data saturation was reached after completion of 5 KIIs with orthopedic surgeons**,** and 15 IDIs with patients and 15 IDIs with caregivers, respectively, as subsequent interviews did not generate substantially new themes relevant to the study objectives. Each KII and IDI lasted for a maximum of 45 minutes**.**

All discussions and interviews were conducted in a convenient setting for participants and were audio recorded with consent to ensure accurate documentation of the information shared. Field notes were also taken during and immediately after FGDs, KIIs, and IDIs to document key observations, contextual information, and relevant non-verbal expressions that complemented the audio-recorded data. No repeat interviews were conducted during the initial qualitative data collection. The transcripts were not returned to participants for comment or correction.

All the four FGDs and 5 KIIs with healthcare providers were conducted in English**,** while most FGDs with community health workers and IDIs with patients and caregivers were conducted in the local language (Luganda), particularly where participants preferred to express their experiences in the local language. All FGDs and interviews were transcribed verbatim. The Luganda interviews were translated into English, while preserving participants’ original accounts and meanings. The translated transcripts were subsequently reviewed against the original audio recordings by an independent researcher to verify their accuracy and completeness and to ensure that no information was omitted or that the original meaning was altered during transcription and translation, to enhance the credibility, accuracy, and trustworthiness of the data**.**

Data collection was conducted by SZ and four trained research assistants (RK, GK, HN, and HK)**,** who had qualifications including Master’s degrees in Public Health (MPH) and Health Services Research (MHSR) with over 10 years of experience in qualitative research data collection. The research team included both male and female researchers. SZ coordinated and supervised the field data collection, including participant recruitment, interviews, focus group discussions, and data management. This study was conducted as part of SZ’s PhD research under the guidance and supervision of EM, FN, NZ, EB, and OK, who held academic ranks ranging from professor, associate professor, and lecturer at the university. The supervisory team had professional and academic interests in public health, orthopedic care, rehabilitation, and post-discharge care**.** The team provided methodological guidance, supported stakeholder engagement, and contributed to the review and interpretation of emerging findings.

Following the initial qualitative data collection, eight co-design group discussion sessions were conducted, comprising four sessions with healthcare providers and four sessions with community health workers**.** The findings from the initial FGDs, KIIs, and IDIs were presented to participants for validation, clarification, prioritization, and further refinement**.** Participants reviewed the identified needs, challenges, and proposed intervention components and provided additional suggestions to improve their relevance, feasibility, acceptability, and practicality. The feedback generated from these sessions informed the modification and refinement of the proposed intervention components.

Following these sessions, the research team engaged study participants in three iterative rounds of back-and-forth consultations to further refine the intervention and develop the final prototype**.** Feedback from each round was incorporated into the subsequent version of the intervention, allowing participants to review, comment on, and further refine the proposed intervention until a final prototype was developed.

### Human-centered design process

The development of the post-discharge intervention followed a HCD approach**,** a participatory and iterative process that engaged healthcare providers, community health workers, patients, and caregivers in identifying post-discharge care needs and developing contextually appropriate solutions. The HCD process comprised five iterative phases: needs assessment and empathy, defining the challenge, ideation, prototype development, and stakeholder review and refinement (S2 Fig)

Phase 1: Needs assessment and empathy. Findings from the initial FGDs, KIIs, and IDIs were used to understand participants’ experiences, needs, and perspectives regarding post-discharge fracture care. Healthcare providers provided insights into discharge planning, rehabilitation, continuity of care, and follow-up challenges; patients and caregivers described their lived experiences, recovery needs, and barriers to care; while community health workers highlighted community-level challenges, referral gaps, and opportunities for strengthening hospital–community linkages. These findings identified key gaps and contextual barriers in post-discharge care and informed the subsequent HCD phases.

Phase 2: Defining the challenge. Findings generated during the needs assessment and empathy phase were reviewed and synthesized to identify the central problem affecting post-discharge recovery. Through facilitated discussions, participants identified the absence of a structured and coordinated post-discharge follow-up system for patients with lower-limb fractures as the central challenge. This problem definition provided a clear focus for generating potential intervention solutions.

Phase 3: Ideation. Healthcare providers and community health workers participated in collaborative discussions to generate potential solutions to the identified post-discharge care gaps. Proposed strategies included strengthening discharge preparation, providing clear information to patients and caregivers, establishing hospital-to-community referral linkages, involving community health workers in follow-up, strengthening caregiver support, improving access to rehabilitation, engaging traditional bone setters in appropriate referral, and increasing community awareness about fracture care and rehabilitation.

Phase 4: Prototype development. The proposed solutions were reviewed, prioritized, and synthesized into a preliminary post-discharge intervention prototype. Prioritization considered the relevance, feasibility, acceptability, practicality, and compatibility of the proposed components with existing health-system and community structures. The resulting prototype integrated facility- and community-based components aimed at strengthening the transition from hospital to community and supporting continuity of care following discharge.

Phase 5: Stakeholder review and refinement. The preliminary prototype was reviewed through eight co-design group discussion sessions, comprising four sessions with healthcare providers and four with community health workers. Participants reviewed the proposed intervention components, clarified roles and delivery processes, and recommended modifications to improve their feasibility, acceptability, cultural appropriateness, and alignment with existing health-system and community structures**.** This was followed by three iterative rounds of consultation, during which feedback from each round was incorporated into subsequent versions of the intervention and returned to stakeholders for further review and refinement. The process resulted in a stakeholder-refined post-discharge intervention prototype package that reflected stakeholder priorities and the operational realities of fracture care in the study setting (S1 File).

### Application of the continuity of care framework

To further strengthen the intervention design, the Continuity of Care framework was adapted to examine gaps in care across the patient’s post-discharge care pathway [36,37]. The framework guided the identification of gaps and intervention opportunities across key stages of the recovery pathway, including hospital care, discharge preparation, hospital-to-community linkage, home recovery, follow-up, and rehabilitation. It also helped the design team examine the roles and coordination of healthcare providers, patients, caregivers, community health workers, and other relevant service providers in supporting continuity of fracture care. In addition, broader health-system and community-level factors, including workforce capacity, financing, governance, service coordination, and community support systems, were considered as potential enablers or barriers to sustained recovery. The identified gaps were subsequently used to inform the development and prioritization of intervention strategies during the co-design process.

### Application of the proctor implementation outcomes framework

The Proctor Implementation Outcomes Framework was then used to guide the prioritization and refinement of the proposed strategies [38]. The intervention components were considered in relation to relevant implementation outcomes, particularly acceptability, appropriateness, feasibility, adoption, and sustainability, to determine whether the proposed strategies could realistically be integrated into routine fracture care. This helped the research team distinguish strategies that were desirable from those that were also practical and implementable within the existing health-system context (Fig 2; S2 File).

### Framework integration and intervention refinement

Thus, the three frameworks served complementary purposes**:** HCD ensured that the intervention remained responsive to stakeholder needs and experiences; the Continuity of Care framework helped identify gaps and opportunities for strengthening care across the post-discharge pathway; and the Proctor Implementation Outcomes framework supported the assessment and prioritization of strategies based on their potential for successful implementation. The iterative stakeholder feedback was subsequently incorporated to refine the intervention prototype and ensure that the final design reflected stakeholder priorities while remaining feasible, contextually appropriate, and aligned with existing health-system structures.

### Data analysis

Data were analyzed using thematic analysis, following the six-step approach described by Braun and Clarke, using NVivo version 15 software [39]. All focus group discussions and interviews were audio-recorded and transcribed verbatim to ensure accuracy and completeness of the information collected. The transcripts were carefully reviewed several times by the research team to achieve familiarity with the data. During the initial stage of analysis, meaningful segments of text were systematically coded, with each code representing a specific idea, experience, or issue related to post-discharge care for patients with lower limb fractures.

Two members of the research team (SZ and RK) independently coded a subset of the transcripts and subsequently compared their coding to assess consistency and identify differences in interpretation. Differences in coding were discussed among the research team, and consensus was reached through discussion and review of the relevant transcript excerpts. A codebook was subsequently developed and iteratively refined throughout the analysis. The codebook included the code name, operational definition, inclusion and exclusion criteria, and illustrative examples of relevant text. New codes were added when concepts emerged from the data that were not adequately captured by the initial coding framework. Similar codes were then compared and grouped together to form broader categories and subthemes representing patient pathways, key barriers, challenges, and potential solutions identified by the participants.

Through this iterative process, major themes emerged that reflected stakeholders’ perspectives on gaps in post-discharge fracture management, barriers to follow-up and rehabilitation, community influences on treatment decisions, and strategies to improve continuity of care after hospital discharge. The final themes were reviewed and refined to ensure that they accurately represented the participants’ views and aligned with the objectives of developing a contextually appropriate post-discharge intervention (S3 File).

### Trustworthiness and rigor

The trustworthiness of the qualitative analysis and reporting process was guided by the principles of credibility, dependability, confirmability, and transferability [40]. Credibility was enhanced through triangulation of data from FGDs, KIIs, and IDIs across different stakeholder groups, including healthcare providers, community health workers, patients, and caregivers; prolonged engagement with the data; comparison of emerging interpretations across stakeholder groups; and the use of representative participant quotations to support the findings. Dependability was strengthened through the use of semi-structured interview guides and systematic documentation of the data collection, coding, and theme-development processes, including maintenance of an audit trail of key analytical and methodological decisions. Confirmability was enhanced by grounding codes and themes in the participants’ accounts, reviewing emerging codes and themes among members of the research team, and checking interpretations against the original transcripts to minimize the influence of individual researcher assumptions. Transferability was supported by providing detailed descriptions of the study setting, participants, sampling procedures, data collection methods, and contextual characteristics to enable readers to assess the applicability of the findings to other settings.

The study was reported in accordance with the Consolidated Criteria for Reporting Qualitative Research (COREQ)**,** including reporting of the research team and reflexivity, study design, participant selection, setting, data collection, data analysis, and reporting of findings [41] (S4 File).

### Confidentiality

Confidentiality was maintained throughout data collection, transcription, analysis, and reporting. Participants were assigned unique identification codes, and their names and other directly identifying information were removed from transcripts and analysis files. Audio recordings, transcripts, and other study materials were stored securely and were accessible only to authorized members of the research team. Electronic files were stored on password-protected devices, while any hard-copy materials were kept in secure locations. Findings were reported using anonymized quotations to prevent individual participants from being identified.

### Ethics approval and consent to participate

Ethical approval for this study was obtained from the Institutional Review Board (IRB) of the Makerere University School of Public Health (Reference: SPH-2022–350) and the Uganda National Council for Science and Technology (UNCST) (Reference: HS4641ES). Administrative permission to conduct the research was granted by the Mulago National Referral Hospital administration and the district authorities of the six districts in Greater Kampala, namely Kampala Capital City, Wakiso, Mukono, Mpigi, Buikwe, and Luweero. Written informed consent was obtained from all participants prior to their enrolment in the study. Participation was voluntary, and participants were informed of the study objectives, procedures, potential risks and benefits, their right to decline participation or withdraw from the study at any time without penalty, and the measures taken to protect their confidentiality.

## Results

### Socio-demographic characteristics of health care providers

A total of 30 healthcare providers participated in the study, comprising 20 males and 10 females. The participants had an overall mean age of 39.2 years. Physiotherapists constituted the largest professional group (n = 9), followed by orthopedic surgeons (n = 5), occupational therapists (n = 5), nurses (n = 5), clinical officers (n = 4), and orthopedic officers (n = 2). Most participants had attained diploma or bachelor’s-level education and had substantial professional experience, with the average years of experience across the professional groups ranging from 5 to 12 years (Table 1).

**Table 1:** Socio-demographic Characteristics of health care providers.

Participant Type	Number	Average Age	Gender Composition		Average Years of Experience	Highest Education Level
			Male	Female		
Orthopedic surgeons	5	41	5		12	Bachelors/ Masters
Orthopedic officers	2	42	2	–	10	Bachelors/ Masters
Physiotherapists	9	40	7	2	8	Diploma/Bachelors
Occupational therapists	5	41	2	3	7	Diploma/Bachelors
Clinical officers	4	37	3	1	7	Diploma
Nurses	5	35	1	4	5	Diploma/Bachelors
**Total**	**30**	**–**	**20**	**10**	**–**	

### Socio-demographic characteristics of community health workers patients and caregivers

A total of 34 community health workers (VHTs), 15 patients, and 15 caregivers participated in the study. Community health workers comprised 18 females and 16 males, patients 6 females and 9 males, and caregivers 13 females and 2 males. The average age was 44 years among community health workers, 40 years among patients, and 36 years among caregivers. Primary education was the most common education level attained across the three participant groups. Most community health workers (29/34) and patients (10/15) were employed. Motorcycle-related injuries were the primary cause of lower-limb fractures among patients (Table 2).

**Table 2:** Socio-demographic characteristics of community health workers, patients and caregivers.

Participants	Community health workers (VHTs)	Patients	Caregivers
**Variable type**			
**Sex**			
Male	16	9	2
Female	18	6	13
**Average age**	44	40	36
**Religion**			
Catholic	17	10	8
Muslim	8	3	3
Protestant	5	2	2
Pentecostal	4		2
**Marital status**			
Married	27	10	11
Not married	7	5	4
**Education level**			
None		1	
Primary	15	7	8
Secondary	13	5	5
Tertiary	6	2	2
**Occupation**			
Employed	29	10	3
Not employed	5	5	12
**Cause of bone fracture**			
Motorcyclist		15	13
Vehicle			2
Total	34	15	15

### Co-designing a post-discharge intervention for patients with lower limb fractures using Human-Centered Design

The analysis generated six major themes describing barriers to effective post-discharge care and one major theme describing co-designed intervention components developed through the HCD process. The themes included: discontinuity of care after hospital discharge; discharge process and communication gaps; social and community influences; use and influence of traditional bone setters; beliefs and misconceptions; health-system structural barriers; and co-designed intervention components (Table 3).

**Table 3:** Themes, subthemes and codes which emerged from the post-discharge intervention development Using HCD.

Theme	Subtheme	Codes
Theme 1: Discontinuity of care after hospital discharge	1.1 Inadequate follow-up systems	Absence of structured follow-up; lack of post-discharge monitoring; unclear follow-up responsibility; weak hospital–community linkage
	1.2 Delayed or missed reviews	Missed appointments; late return to hospital; poor appointment tracking; inadequate reminders
	1.3 Transport and financial barriers	High transport costs; lack of transport; distance to facility; loss of income; competing household expenses
	1.4 Physical mobility constraints	Difficulty walking; dependence on caregivers; inability to travel independently; pain and limited movement
Theme 2: Discharge process and communication gaps	2.1 Limited discharge counselling	Short counselling time; busy health workers; inadequate explanation; rushed discharge
	2.2 Incomplete discharge information	Missing care instructions; unclear rehabilitation guidance; inadequate information on warning signs; unclear review dates
	2.3 Poor understanding and retention	Poor comprehension; language barriers; information overload; misunderstanding instructions; forgotten instructions
	2.4 Caregiver communication gaps	Different caregiver at home; information not transferred; caregiver not present at discharge; loss of discharge information
Theme 3: Social and community influences	3.1 Community and family influence	Family advice; peer influence; community recommendations; pressure to change treatment
	3.2 Peer recovery narratives	Stories of rapid recovery; comparison with others; imitation of previous treatment experiences
	3.3 Fracture treatment misconceptions	Misconceptions about POP; beliefs about delayed healing; misunderstanding pain and healing; fear of hospital treatment
	3.4 Spiritual and cultural beliefs	Spiritual explanations; traditional beliefs; ritual healing; cultural interpretations of fracture recovery
Theme 4: Use and influence of traditional bone setters	4.1 Accessibility and convenience	Proximity; flexible opening times; easy access; community availability
	4.2 Financial affordability and flexibility	Lower perceived cost; flexible payment; deferred payment; negotiable fees
	4.3 Trust and perceived personalized care	Relational trust; familiarity; cultural acceptance; personalized attention; perceived effectiveness
	4.4 Treatment practices and complications	Premature POP removal; manipulation of fractures; inappropriate immobilization; malunion; worsening pain
Theme 5: Health-system structural barriers	5.1 Workforce constraints	Staff shortages; inadequate orthopedic staff; limited physiotherapists; single-provider departments; workload
	5.2 Resource limitations	Limited airtime; inadequate outreach transport; insufficient equipment; limited rehabilitation resources
	5.3 Centralization of fracture services	Dependence on referral hospitals; long travel distances; limited services at lower facilities; referral delays
	5.4 Weak coordination and referral systems	Poor referral communication; unclear roles; weak facility–community coordination; limited information sharing
Theme 6. Beliefs and misconceptions	6.1 Fracture Treatment Misconceptions	Misunderstanding fracture healing, belief that hospital treatment is insufficient; incorrect treatment beliefs
	6.2 POP Myths	POP causes itching, POP causes lice; POP delays healing
	6.3 Healing Speed Misinterpretation	Pain stopped so fracture is healed, less pain means complete healing; feels better therefore stops care
	6.4 Spiritual Explanations	Healing attributed to spiritual forces; ritual treatment; spiritual explanation for complications
Theme 7: Co-designed intervention components	7.1 Structured discharge system	Standardized discharge checklist; discharge education package; contact documentation; scheduled review date
	7.2 Facility–community linkage	VHT notification; referral to community health workers; discharge alerts; community follow-up
	7.3 VHT capacity strengthening	Training VHTs; POP monitoring; basic rehabilitation support; danger-sign recognition; referral triggers
	7.4 Appointment reminder and follow-up system	Telephone reminders; SMS reminders; follow-up calls; appointment tracking
	7.5 Traditional bone setter engagement and referral	Defined referral boundaries; collaboration; referral pathways; communication with health facilities
	7.6 Decentralized follow-up services	Follow-up at lower-level facilities; community-based review; outreach services; reduced travel burden
	7.7 Policy and financing support	Staffing advocacy; sustainable financing; outreach funding; integration into routine services

### Theme 1: Discontinuity of care after hospital discharge

Several participants described significant gaps in continuity of care between hospital discharge and community recovery. Healthcare providers reported that health facilities lack structured systems to monitor patients once they leave the hospital. As a result, many patients failed to return for scheduled follow-up appointments or returned after long delays. Several barriers contributed to missed follow-up visits. Participants reported that transport costs and long travel distances prevented many patients from attending review appointments. In addition, mobility limitations following injury made it difficult for patients to travel independently to health facilities for follow-up care. These factors resulted in many patients continuing recovery without adequate clinical monitoring.

*“But here, when patient is discharged and goes back to his/her home, there is no support or follow-up from the hospital to monitor the patient’s recovery”* ***(KIIs-Health Worker-Kampala)***

*“When patients go back home after discharge, it is difficult for us to know how they are progressing because there is no clear follow-up arrangement. Sometimes we only hear about them when they develop a problem or come back to the health facility.” **(FGD-CHW-Luweero, P2)***

*“After I was discharged, nobody followed me up to know how I was doing. Going back to the hospital was difficult because I had no money for transport, so I stayed at home”* ***(IDI_Patient_Wakiso).***

*“After we came back home from the hospital, there was no one to follow up with us. We were not sure what we were supposed to do at home or when to take the patient back for review.* ***(IDI-Caregiver-Mpigi)***

### Theme 2: Discharge planning and information disclosure

Several participants highlighted weaknesses in the discharge process, particularly regarding communication of discharge instructions. Due to the high patient volumes and limited staffing in orthopedic units, healthcare providers often have insufficient time to provide comprehensive counseling before discharge. Participants also reported that discharge instructions are sometimes incomplete or poorly understood by patients and caregivers. In some cases, the caregiver at discharge counseling is not the same individual responsible for providing care at home. Participants opined that this discontinuity leads to loss of critical information related to medication use, mobility restrictions, plaster care, and follow-up appointments.

*“There are many patients waiting to be attended to, so sometimes we do not have enough time to explain everything to the patient and caregiver before discharge.”* **(FGD-Health Worker-Mukono, P5).**

*“Sometimes when we visit patients at home, the caregiver tells us that they were not given enough information at discharge. They may know that the patient has to return for review, but they are not clear about the other instructions.” **(FGD-CHW-Mpigi, P1)***

*“At the hospital, they did not give us enough instructions at discharge because there was little time to ask questions, as many patients were waiting. When we reached home, I did not know how to care for the plaster or when to return for review.”* ***(IDI_ Caregiver_Wakiso)***

*“They explained some things to us at the hospital, but there was little time. When I reached home, I could not remember everything they had told me about taking care of the plaster and when to come back.” **(IDI-Patient-Buikwe)***

### Theme 3: Social and community influence

Several health care providers and community health workers emphasized that decisions about fracture care after hospital discharge are strongly influenced by social networks within the community. Advice from family members, friends, and neighbors often shape patient behavior during recovery. Participants reported that some community members encourage patients to remove plaster casts prematurely or discontinue follow-up visits based on personal recovery experiences. These community beliefs often conflict with professional medical advice and influenced patients’ treatment choices.

*“Some patients come back and tell us that their friends or relatives advised them not to continue with hospital treatment because they had recovered after being treated by a traditional bone setter. They tend to compare their experience with what they have been told by people in the community.”* **(KIIs-Health Worker-Kampala).**

*“What usually leads patients to seek care from traditional bone setters in the village is the belief that healing in hospitals takes a long time. After discharge, when a POP cast has been applied, caregivers often suggest taking the patient to a traditional bone setter to remove it, believing that it will make it easier to set the bones.”* ***(FGD CHW_Mukono, P1)***

*“People in the community would tell us different things about the plaster. Some said we should remove it when the pain became less, while others advised us to take the patient to a bone setter. It was difficult to know which advice to follow.” **(IDI-Caregiver-Buikwe)***

*“When I went back home, my relatives and neighbors told me that I should go to a traditional bone setter because they believed I would heal faster there. They said the hospital treatment takes a long time, so I followed their advice.” **(IDI-Patient-Kampala)***

### Theme 4: Influence of traditional bone setters

Healthcare providers reported that some patients seek treatment from traditional bone setters after hospital discharge. Several factors contributed to this practice, including proximity of traditional practitioners, flexible payment arrangements, and strong cultural trust in traditional healers. However, healthcare providers reported that seeking care from traditional bone setters sometimes results in complications because of premature removal of plaster casts, inappropriate manipulation of injured limbs, and improper immobilization practices. These practices increased the risk of delayed healing, malunion, persistent pain, and long-term disability

*“We have seen patients coming back after visiting traditional bone setters with the plaster already removed or the limb manipulated. By the time they return, the fracture may not be in the correct position and they complain of continued pain.” **(FGD-Health Worker-Luweero, P4)***

*“Some patients leave the hospital and go to traditional bone setters because they are nearby, trusted in the community, and allow flexible payment.”* (***FGD-CHW-Buikwe, P2)***

*“We decided to take him to the bone setter because the hospital was far and we did not have enough money for transport. The bone setter was nearby, and he told us that we could pay when we got the money.” **(IDI-Caregiver-Mukono)***

*“When I was still having pain after leaving the hospital, my relatives advised me to go to traditional bone setter there because they believed he could make me heal faster.” **(IDI-Patient-Wakiso)***

### Theme 5: Health system structural limitations

Health care providers identified several structural challenges within the health system that limit effective post-discharge care. These included shortages of orthopedic and physiotherapy personnel, with some departments being managed by only one therapist. Participants also reported limited operational resources to support patient follow-up activities. It was noted that many facilities lack funding for patient reminder calls, and there were no mechanisms to facilitate outreach visits. In addition, fracture services were largely centralized at referral hospitals, requiring patients to travel long distances to access follow-up care. Participants further noted that weak referral systems and limited of integration of rehabilitation services at lower-level health facilities constrained continuity of care after discharge.

*“In the hospital, we have very few physiotherapy staff compared to the number of patients who need rehabilitation. Sometimes one therapist has to attend to many patients, making it difficult to provide follow-up to everyone.”* **(KIIs-Health Worker-Kampala).**

*“We do not have resources specifically for following patients after discharge. For example, there is no funding for making reminder calls or visiting patients in the community.” **(FGD-Health Worker-Luweero, P2)***

*“There is no arrangement for us to visit patients who have been discharged. We may not know that someone has gone home with a fracture, there is no specific programme to follow up such patients in the community.” **(FGD-CHW-Mpigi, P6)***

*“The patient was supposed to return to the hospital for review, but the hospital is far from where we live. Sometimes we had to wait until we had enough money for transport before going back.” **(IDI-Caregiver, Buikwe)***

### Theme 6: Beliefs and misconceptions about fracture treatment

Participants described several misconceptions about fracture healing and treatment within the community. Some community health workers mentioned that plaster casts cause itching, get infested with lice, and cause delayed healing, leading some patients to remove the casts prematurely. Other misconceptions included mis-interpreting pain relief as evidence that the fracture had completely healed, which sometimes results in early discontinuation of treatment. In some cases, fracture healing was also attributed to spiritual or ritual explanations, rather than hospital-based treatment.

*“We sometimes receive patients who have removed the plaster because they believe that once the pain has stopped, the fracture has healed. They do not always understand that the bone may still need time to heal.” **(FGD-Health Worker-Luweero, P5)***

*“Some people told me that the fracture was not only a problem and that I needed to seek help through traditional or spiritual healers for the bone to heal properly.” **(IDI-Patient-Luweero)***

*“Some people in the community believe that the plaster causes itching and can even get lice. Because of this, some patients remove the plaster before the time they were told to return to the hospital.” **(FGD-CHW-Mpigi, P1)***

*“People in the community tell us different things about fractures. Some say that the plaster is not necessary and that the bone can heal through other means, so sometimes you are not sure which advice to follow.” **(IDI-Caregiver-Buikwe)***

### Theme 7: Co-designing intervention components

Participants collaboratively proposed several interventional strategies aimed at addressing the identified barriers and strengthening continuity of care after hospital discharge.

### Subtheme 1: Structured discharge system

Many healthcare providers and community health workers proposed the development of a structured discharge system to standardize patient counseling and discharge procedures. This would include a discharge checklist to ensure that healthcare providers consistently communicate essential information regarding home care, medication use, mobility precautions, and follow-up appointments. The system would also involve documenting contact information for patients, caregivers, and community health workers to facilitate communication after discharge.

*“We need a checklist that guides us on what information should be given to every patient before discharge. This would help ensure that important things such as medication, plaster care, movement, and the next appointment are explained.” **(KIIs-Health Worker-Kampala)***

*“When the patient goes home, we need to know where they stay and how to contact the patient or caregiver. If the community health worker is also identified at discharge, it would be easier to follow up with the patient.” **(FGD-CHW,Buikwe, P4)***

*“They should explain everything clearly before we leave and give us the instructions to take home, because sometimes you forget what you were told at the hospital.” **(IDI-Patient-Mukono)***

*“It would help if we were given something written showing the medicines to take, how to care for the plaster, what to avoid, and when we should return to the hospital.” **(IDI-Caregiver-Luweero)***

### Subtheme 2: Community linkage mechanism

Several healthcare providers and community health workers recommended the establishment of a formal linkage system between health facilities and community health workers. Under this system, discharged patients would be connected to community health workers who can provide follow-up support and monitor recovery within the community.

*“Before the patient leaves the hospital, there should be a clear arrangement to link them with a community health worker in their area. The community health worker can then know the patient and follow up with them after discharge.” **(FGD-Health Worker-Mpigi, P3)***

*“If we are informed when a patient is discharged, we can visit them at home, see how they are progressing, and remind them about their appointments and other instructions given at the hospital.” **(FGD-CHW-Luweero, P1)***

### Subtheme 3. Capacity strengthening for community health workers

Healthcare providers and community health workers suggested training of community health workers, particularly VHTs, to support post-discharge fracture care. Training would focus on recognizing plaster cast complications, monitoring patient recovery, identifying warning signs, and referring patients back to health facilities when necessary.

“*The VHTs need to be trained on what to look for when they visit patients with fractures, including problems with the plaster, swelling, increasing pain, or other signs that require the patient to return to the hospital.”* **(FGD-Health Worker- Luweero, P2)**

*“If we are trained, we can follow up patients at home and identify problems early. We can also know which problems we can advise on and which ones require referral to the health facility.” **(FGD-CHW-Mukono, P2)***

### Subtheme 4: Appointment reminder and follow-up system

Healthcare providers and community health workers emphasized the need for a structured appointment reminder and follow-up system to strengthen continuity of care after hospital discharge. The proposed system would include telephone reminders, SMS reminders, follow-up calls, and appointment tracking to remind patients about scheduled reviews, identify those who miss appointments, and facilitate timely follow-up. Healthcare providers felt that such a system could improve communication between patients and healthcare providers and reduce loss to follow-up, particularly among patients recovering at home.

“*Sometimes patients are given a return date, but they do not come back. If we had a system for calling or sending them a message before the appointment, it would help us follow them up*.” **(KIIs-Health Worker-Kampala)**

“*If we know the date when the patient is supposed to return to the hospital, we can remind them by phone and also follow up with those who have missed their appointment.*” **(FGD-CHW-Mpigi, P5)**

### Subtheme 5: Engagement of traditional bone setters

Several healthcare providers and community health workers recommended developing clear referral pathways and communication channels between traditional bone setters and healthcare providers to ensure that patients who initially seek traditional care can be referred early for medical evaluation when necessary. Such engagement was viewed as a practical approach to improve patient safety, minimize harmful practices, and strengthen coordination between community-based traditional providers and the formal healthcare system.

*“We should not ignore the traditional bone setters because many patients in the community go to them. Instead, we need to engage them and establish a clear pathway for them to refer patients to the hospital when they identify a problem.”* ***(FGD- Health Worker-Mpigi, P1)***

*“Traditional bone setters are known in the community and people trust them. If there was a link between them and the health facility, they could refer patients to the hospital when they see something that needs medical attention.” **(FGD-CHW-Buikwe, P5)***

### Subtheme 6: Decentralization of follow-up services

Several healthcare providers recommended expanding fracture review services to lower-level health facilities to reduce travel barriers and improve accessibility. Decentralizing follow-up care would enable patients to receive routine monitoring closer to their homes.

*“Patients should not have to travel all the way to the referral hospital for every review. Some of the routine follow-up services can be provided at lower-level health facilities closer to where they live.” **(KII-Health Worker-Kampala)***

*“Many patients have difficulties travelling back to the hospital after discharge. If some follow-up services are available at a nearby health center, it would be easier for them to continue with their reviews.” **(FGD-CHW-Mukono, P3)***

### Subtheme 7: Policy and financing advocacy

Several healthcare providers and community health workers emphasized the need for policy engagement to strengthen rehabilitation services and address workforce shortages in orthopedic and physiotherapy care. Advocacy efforts would focus on improving funding for follow-up systems, communication mechanisms, and staffing to support long-term recovery of fracture patients.

*“So, the policy has to be put in place by the government to guide how fracture patients are cared for after discharge, and also there should be available funds to support their treatment and follow-up care after discharge”* ***(FGD-Health Worker-Luwero, P3).***

*“If VHTs are expected to follow up patients with fractures in the community, they need some support to do this work. Without resources for transport and other follow-up activities, it is difficult for us to visit patients regularly in their homes.” **(FGD-CHW-Buikwe, P6)***

*“It would be helpful if there was some support for patients after discharge. Sometimes we need to return to the hospital for rehabilitation, but transport and other costs make it difficult.”* ***(IDI-Caregiver-Mpigi)***

### Continuity of care framework for post-discharge intervention development using human-centered design

To operationalize the HCD approach, the design team adopted a *Continuity of Care Framework [36,37]* to guide the identification of gaps and intervention opportunities across the patient recovery pathway, from hospitalization to long-term community recovery. The framework emphasizes coordinated care through stages including hospital care, discharge preparation, hospital community linkage, home recovery, follow-up, and rehabilitation. It highlights key challenges while identifying the roles of health workers, caregivers, community health workers, and patients in supporting recovery. The framework also incorporates broader health system enablers including workforce capacity, financing, governance, and community support systems to promote patient-centered and sustainable recovery after lower limb fractures The Continuity of Care Framework guided the identification of gaps and intervention opportunities across the recovery pathway (Fig 1).

**Fig 1:**
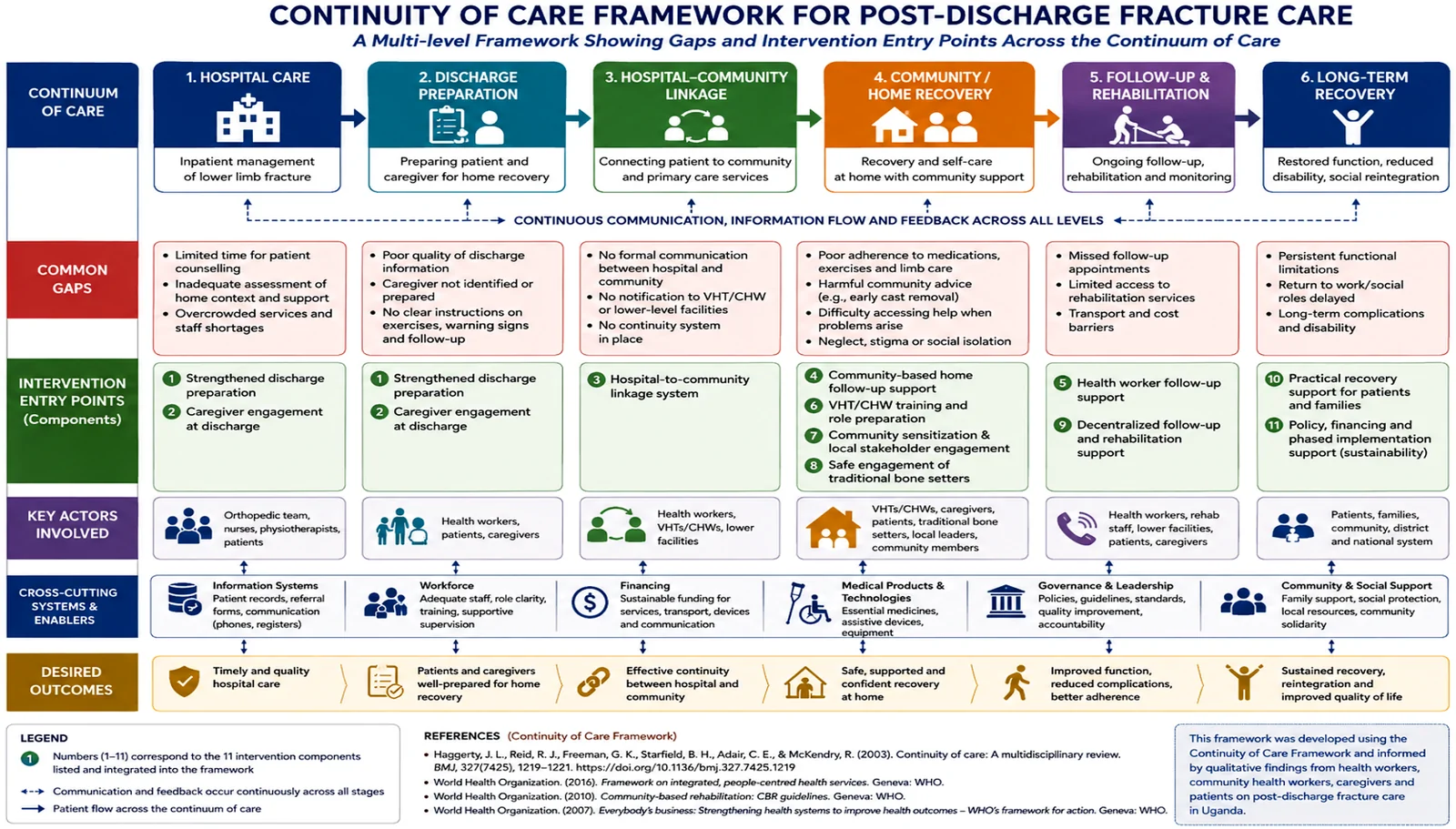
Continuity of Care Framework adapted for post discharge intervention development.

### Feasibility and implementation framework linking proposed interventions to existing programs in Uganda

Participants, including healthcare providers and community health workers demonstrated that the proposed post-discharge fracture care intervention can be integrated into existing health and community systems in Uganda, particularly the Community Health Extension Worker (CHEW) program and the Village Health Team (VHT) strategy, which are designed to strengthen community-based healthcare delivery, referral systems, and continuity of care. Participants further identified several existing health programs and policies that are well aligned with the proposed intervention, including Uganda’s Primary Health Care strengthening agenda and rehabilitation initiatives that seek to decentralize healthcare services and strengthen community-level support for patients with long-term recovery needs (Table 4).

**Table 4:** Feasibility and implementation framework linking proposed interventions to existing programs in Uganda.

Proposed Intervention Component	Existing Program/Policy in Uganda	How the Intervention Can Be Integrated
Strengthened discharge preparation	Uganda Ministry of Health Integrated People-Centered Care approach	Standardized discharge counselling tools can be incorporated into routine inpatient and rehabilitation services to improve continuity of care.
Caregiver engagement at discharge	Family-centered care approach under hospital quality improvement initiatives	Caregivers can formally be included in discharge planning, education, and home recovery preparation.
Hospital-to-community linkage system	Community Health Extension Worker (CHEW) Programme and Village Health Team (VHT) Strategy	Referral and follow-up communication systems can be integrated into CHEW and VHT community linkage roles to support continuity of care after discharge.
Community-based and home follow-up support	Community Health Extension Worker (CHEW) Programme; Village Health Team (VHT) Programme	CHEWs and VHTs can conduct home visits, monitor recovery, provide health education, and support referral back to health facilities when complications arise.
Health worker follow-up support	Primary Health Care (PHC) strengthening initiatives	Follow-up calls, appointment reminders, and decentralized patient monitoring can be integrated into PHC outreach and rehabilitation services.
VHT/CHW training and role preparation	CHEW National Training Guide and Community Health Worker capacity-building programmes	Fracture recovery and rehabilitation modules can be incorporated into existing CHEW/VHT training packages and supportive supervision systems.
Community sensitization and local stakeholder engagement	Community mobilization structures involving LC1 leaders, religious leaders, and community dialogues	Existing community engagement platforms can be used to increase awareness on safe fracture care, rehabilitation, and harmful practices.
Engagement of traditional bone setters	Ministry of Health community engagement and referral strengthening strategies	Traditional bone setters can be engaged by sensitizing them to recognize danger signs and encourage early referral of fracture patients to formal health facilities.
Decentralized follow-up and rehabilitation support	Uganda Rehabilitation and Assistive Technology initiatives; Primary Health Care strengthening programmes	Selected lower-level health facilities can be strengthened to provide basic rehabilitation and follow-up closer to patients’ homes.
Patients and family’s recovery support	Social protection and community support programmes	Linkages to district social support services, disability support programmes, and community welfare structures can improve recovery support.
Policy, financing, and phased implementation support	CHEW National Policy and Health Sector Development Plans	The intervention can be piloted and gradually integrated into existing community health and rehabilitation systems under Ministry of Health structures.

### Prioritization of intervention components for policy formulation in Uganda

The prioritization process was informed by the Proctor Implementation Outcomes Framework which emphasizes implementation outcomes such as feasibility, acceptability, appropriateness, adoption, fidelity, penetration, scalability, sustainability and resource requirements when assessing health interventions [38]. In addition, the study considered the practicality of integrating interventions within existing Uganda Ministry of Health structures, including the Community Health Extension Worker (CHEW) Programme, Village Health Team (VHT) strategy, and integrated people-centered care approaches.

Although several intervention components were identified during the co-design process, not all were prioritized for immediate policy recommendation and implementation. Some interventions were excluded because they required substantial financial resources, additional workforce, infrastructure development, or major policy reforms that may not be immediately feasible within the current Uganda health system context.

Other interventions demonstrated lower sustainability, limited scalability, or dependence on long-term multisectoral coordination and external funding. In addition, certain interventions such as decentralized rehabilitation services and extensive home follow-up support would require significant expansion of rehabilitation infrastructure, staffing, transport facilitation, and operational funding, which may present implementation challenges in low-resource settings. Therefore, the prioritization process focused on interventions that could be integrated more easily into existing Ministry of Health structures, implemented with relatively fewer additional resources, and sustained within the current community and primary healthcare systems

During the prioritization process, two interventions were considered to be most feasible and appropriate for recommendation to policy makers based on their high feasibility, acceptability, sustainability, scalability, and compatibility with existing health system structures in Uganda including; (1) strengthened discharge preparation using standardized discharge tools and (2) strengthened hospital to community linkages through community health workers The Proctor Implementation Outcomes Framework guided the prioritization of the proposed intervention strategies (Fig 2).

**Fig 2:**
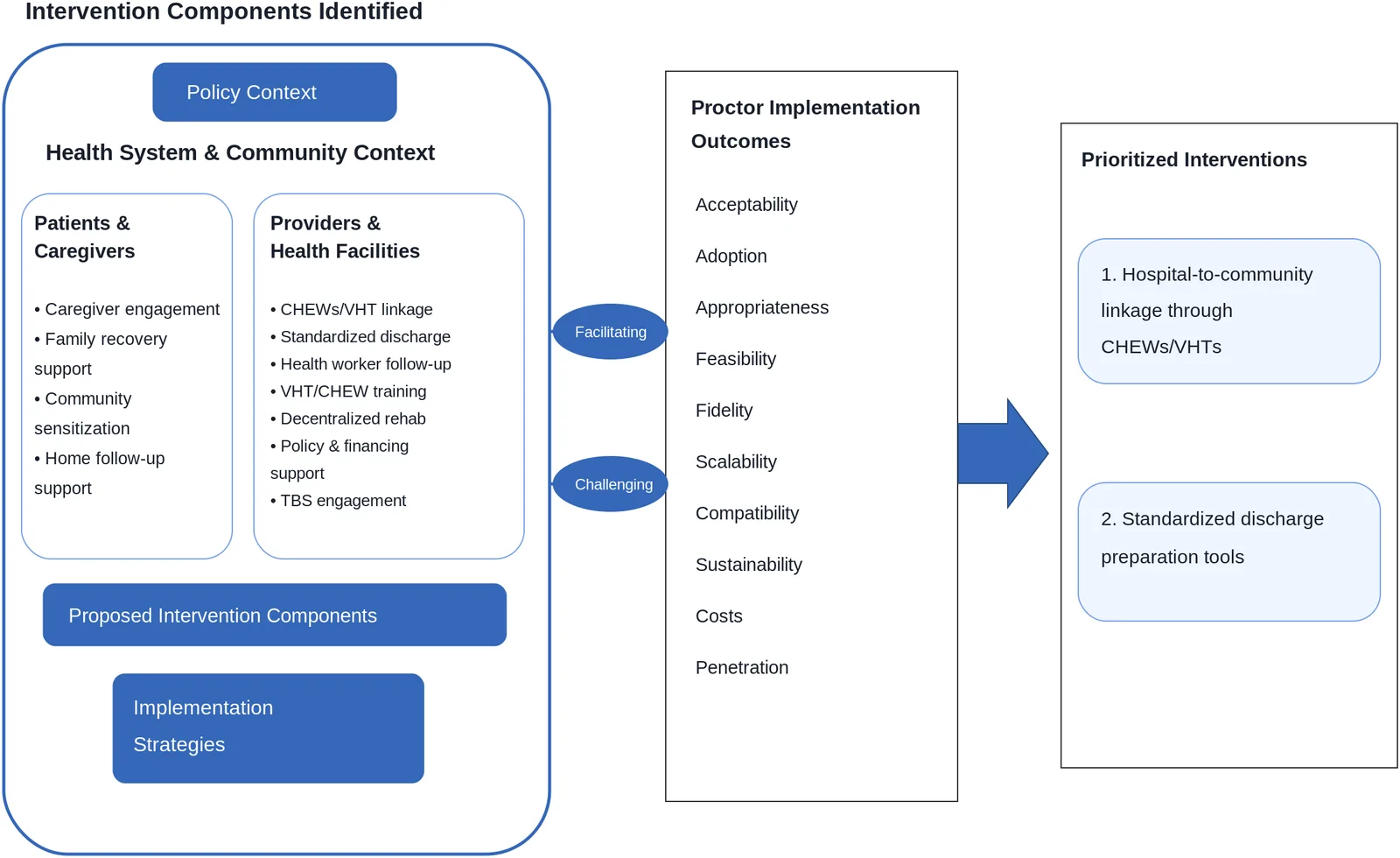
Proctor Implementation Outcomes Framework for prioritizing intervention strategies.

## Discussion

This study used a HCD approach to co-develop a post-discharge intervention aimed at improving mobility recovery among patients with lower limb fractures in Greater Kampala, Uganda. The findings revealed multiple interconnected barriers affecting continuity of care after hospital discharge, including discontinuity of care, weaknesses in the discharge process, strong community influences on treatment decisions, reliance on traditional bone setters, structural health system limitations, and widespread misconceptions about fracture treatment. Through a participatory process involving participants collaboratively proposed several intervention components aimed at strengthening continuity of care between health facilities and communities. Human-Centered Design approaches have increasingly been applied in global health to develop interventions that are responsive to user needs and contextual realities within health systems [25,28,29].

The HCD process specifically contributed to the development of the proposed intervention by providing a participatory and iterative pathway through which healthcare providers and community health workers, as well as patients and caregivers, collaboratively shared their experiences and perspectives on post-discharge care, moving beyond identifying problems to co-generating, prioritizing, and refining solutions**.** This process may not have been readily achieved through conventional qualitative needs assessment, which primarily focuses on identifying and describing problems rather than systematically engaging stakeholders in the iterative development and refinement of intervention solutions [42,43]. Thus, the contribution of HCD was not simply the identification of problems and needs, but rather the iterative and participatory process through which stakeholder experiences and needs were translated into specific, contextually responsive, and potentially implementable intervention components [44].

One of the major findings of this study was the lack of a structured and coordinated post-discharge follow-up system for patients with lower limb bone fractures. Participants reported that once patients left the hospital, there were limited mechanisms to ensure that they returned for review appointments or received adequate support during recovery. Similar challenges have been reported in other low- and middle-income settings, where weak referral systems and limited follow-up contribute to poor continuity of trauma care and delayed recovery among fracture patients [19,45–47]. Missed follow-up appointments in this study were largely attributed to transportation barriers and mobility limitations, highlighting the need for decentralized follow-up systems and community-based monitoring mechanisms. Community-based follow-up approaches involving community health workers have been shown to improve continuity of care and patient outcomes in resource-limited settings [24,48,49]. Therefore, the health system should establish structured post-discharge care for patients with bone fractures through regular follow-ups and by integrating community health workers into home-based rehabilitation service provision.

The study also identified weaknesses in the discharge process, particularly patient counseling and information disclosure. Healthcare providers noted that heavy workloads often limit the time available for comprehensive discharge education, and in some cases the caregiver present at discharge is not the same person responsible for providing care at home. Poor discharge communication has been widely documented as a contributor to treatment non-adherence, medication errors, and poor recovery outcomes among patients after hospital discharge [50–52]. Implementing structured discharge systems, including standardized counseling tools and checklists, may therefore improve patient understanding of post-discharge care and promote adherence to treatment recommendations. Previous studies have shown that structured discharge planning significantly improves patient engagement and recovery outcomes after hospitalization [20,53,54] The Health system should implement standardized discharge counselling tools and checklists to improve patient and caregiver understanding of post-discharge care, rehabilitation, medications, and follow-up requirements.

Community and social influences were also found to significantly affect treatment decisions after discharge. Participants described how family members, peers, and community narratives often shape patient behavior during recovery. Our findings suggest that advice from community members sometimes encourages premature removal of plaster casts or discontinuation of follow-up visits. This highlights the important role of social networks in shaping health-seeking behaviors, particularly in community-based recovery contexts. Social and cultural factors have been shown to strongly influence adherence to medical treatment and rehabilitation practices in many low-resource settings [55–57]. Strengthening community engagement and patient education may therefore be essential for improving adherence to fracture care recommendations.

The study further highlighted the continued utilization of care from traditional bone setters. Participants noted that patients often seek care from traditional bone setters due to accessibility, cultural familiarity, and flexible payment arrangements. However, healthcare providers expressed concern that such practices often result into improper immobilization, delayed presentation to hospitals, resulting into complications such as malunion or nonunion, infections, joint stiffness, deformities, and sometime limb amputation. Previous studies across Sub-Saharan Africa have similarly documented the widespread use of traditional bone setters in fracture care [58–62]. Rather than excluding traditional practitioners, participants suggested engaging them through sensitization and referral linkages to improve coordination between traditional and health system. Collaborative approaches between traditional and formal health providers have been recommended as a strategy for reducing complications while respecting local cultural health practices [63]. The health system should engage traditional bone setters through sensitization and awareness programs to be able recognize danger signs and ensure timely referral of patients with fractures to hospitals.

Structural challenges within the health system also emerged as key barriers to effective post-discharge fracture care. Health care providers identified shortage of orthopedic and physiotherapy personnel, limited resources for patient follow-up, and the centralization of orthopedic services at referral hospitals. These structural constraints are consistent with broader health system challenges documented in trauma care across low-resource settings [47,64,65]. The World Health Organization has emphasized that rehabilitation services remain underdeveloped in many countries despite the growing burden of injuries and musculoskeletal conditions [66]. Addressing these gaps requires policy-level efforts to strengthen rehabilitation services, expand the orthopedic workforce, and improve resource allocation for follow-up care.

The HCD process enabled stakeholders to co-develop practical intervention strategies tailored to the local context. Proposed components included the introduction of structured discharge checklists, improved documentation of patient and caregiver contact information, stronger linkage systems between health facilities and community health workers, training of community health workers to support fracture follow-up, and phone-based reminder systems for review appointments. Participants also recommended decentralizing follow-up services to lower-level health facilities to reduce transportation barriers. These proposed strategies align with broader global recommendations emphasizing strengthening continuity of post-discharge care and improving access to rehabilitation services, particularly in low-resource settings where rehabilitation services are very limited [66–68].

Though the present study was conducted in Greater Kampala, the findings may be applicable to other low-resource settings with similar sociodemographic characteristics and health-system contexts like Uganda**.** The challenges identified, including discontinuity of post-discharge care, inadequate discharge preparation and information transfer, limited access to rehabilitation services, transport and financial barriers, reliance on traditional bone setters, inadequate follow-up mechanisms, limited health-system resources, and inadequate support for community health workers**,** have been reported in other previous studies conducted in low- and middle-income countries [47,62,64]. The proposed strategies for strengthening discharge preparation, improving facility–community linkages, and engaging community health workers address common challenges in post-discharge fracture care and may therefore be applicable in comparable settings. The participatory nature of the HCD approach further provides an opportunity to adapt these intervention components to local community needs, available resources, healthcare structures, and cultural practices, supporting their application across similar low-resource settings.

Overall, this study demonstrates that the HCD approach is a potentially useful framework for developing contextually appropriate interventions within complex health systems. By actively involving healthcare providers and community stakeholders in the design process, the study identified practical solutions that reflect real-world challenges and opportunities within the Ugandan healthcare system. Participatory approaches such as HCD have been shown to enhance intervention acceptability, feasibility, and sustainability of health programs [25,69]. The proposed co-designed intervention integrates facility-based and community-based components, which may help improve continuity of care and support mobility recovery among patients with lower limb fractures.

Future research should evaluate the feasibility, effectiveness, and scalability of the proposed intervention in improving follow-up adherence, reducing complications, and enhancing functional recovery outcomes among fracture patients. Such implementation research will be important for informing policy and strengthening post-discharge care systems for trauma patients in Uganda and other similar settings.

### Strength and limitation of the study

The major strength of this study was the use of a HCD approach, which actively involved healthcare providers, community stakeholders, patients and caregivers in identifying challenges and co-designing practical intervention strategies. This participatory approach enabled the development of contextually relevant recommendations that reflect the realities of post-discharge fracture care within the Ugandan health system and other low resource settings with similar sociodemographic profile. In addition, the inclusion of multiple stakeholders provided diverse perspectives that enriched understanding of the barriers and opportunities for improving continuity of care.

The main limitation of this study is that the proposed intervention framework was not piloted or validated within the current study; therefore, its feasibility and effectiveness remain to be established through future implementation research.

## Conclusion

This study applied a HCD approach to co-develop a post-discharge intervention aimed at improving continuity of care and mobility recovery among patients with lower limb fractures in Greater Kampala, Uganda. The findings revealed multiple barriers affecting post-discharge fracture care, including discontinuity of follow-up, weaknesses in the discharge process, strong social and community influences on treatment decisions, influence of traditional bone setters, structural limitations within the health system, and widespread misconceptions about fracture treatment. These challenges contribute to missed follow-up appointments, poor adherence to treatment recommendations, and delayed recovery among fracture patients.

Through the participatory design process, healthcare providers, community health workers, patients and caregivers collaboratively identified feasible strategies to address these challenges. The proposed intervention integrates facility-based and community-based components, including structured discharge systems, improved communication and documentation, community health worker involvement in follow-up care, reminder mechanisms for review appointments, decentralization of fracture follow-up services, and engagement with traditional bone setters. The HCD process ensured that the intervention reflects the practical realities of the local health system and the needs of both patients and healthcare providers.

## Supporting information

S1 Fig Participant recruitment flow diagram. (TIF)

S2 Fig Human-centered design process. (TIF)

S1 File Intervention prototype package. (DOCX)

S2 File Participant-informed intervention prioritization matrix. (DOCX)

S3 File Codebook for co-designing a post-discharge intervention. (DOCX)

S4 File COREQ (Consolidated Criteria for Reporting Qualitative Research) checklist. (DOC)
