# Supplementary material for: Co-designing a post-discharge intervention to improve mobility recovery among patients with lower limb fractures using human-centered design in greater Kampala, Uganda
Source: PLoS One. 2026 Nov 10;21(11):e0360232. doi: 10.1371/journal.pone.0360232 (PMC13750591; doi:10.1371/journal.pone.0360232)
Supplement: Supplementary file 1 [file pone.0360232.s003.docx]

**INTERVENTION PROTOTYPE PACKAGE**

**Stakeholder Refined Post Discharge Mobility Recovery Intervention**

**For Patients with Lower Limb Fractures in Greater Kampala, Uganda**

**1. Package Overview**

The intervention package translates stakeholder co-design priorities into a structured post-discharge intervention to improve continuity of care and mobility recovery among patients with lower limb fractures in Greater Kampala, Uganda. It comprises **two prioritized core components: (1) standardized discharge preparation and (2) hospital-to-community linkage through CHWs/VHTs/CHEWs,** supported by community/home follow-up, caregiver involvement, reinforcement of the prescribed mobility and rehabilitation plan, referral and escalation, and facility–community communication. **Standardized discharge preparation** ensures that patients and caregivers leave hospital with clear and actionable information on fracture recovery, mobility precautions, prescribed exercises or activities, walking-aid use where applicable, aftercare, warning signs and scheduled follow-up. A standardized discharge checklist, patient/caregiver information sheet and understanding check are used to promote consistent delivery**. Hospital-to-community linkage** establishes a defined transition from hospital to home/community by connecting eligible patients with an existing CHW/VHT/CHEW before or immediately after discharge. Community workers reinforce the facility-provided recovery plan, assess mobility progress and barriers, support caregivers, identify warning signs and facilitate referral to appropriate clinical or rehabilitation services when required. They do not independently prescribe rehabilitation or alter clinical treatment. The package therefore provides a **continuity-of-care pathway from hospital discharge to community-based support,** using existing health-system structures rather than creating a parallel workforce. It is a **stakeholder-refined intervention prototype**, not yet formally usability-tested or field-tested, and is intended for technical review, final refinement, feasibility/pilot testing and subsequent effectiveness evaluation.

**2. Package structure and components**

| **Intervention layer** | **Intervention elements** | **Purpose** |
| --- | --- | --- |
| CORE 1 | Standardized discharge preparation | Ensure every eligible patient and caregiver receives clear, actionable information on mobility recovery, prescribed exercises/activities, aftercare, warning signs and follow-up before discharge. |
| CORE 2 | Hospital-to-community linkage | Establish a clear and documented link between the hospital and the patient’s home/community to support continuity of post-discharge care. |
| SUPPORT 1 | CHW/VHT/CHEW follow-up | Reinforce the facility-provided recovery plan, assess progress and barriers, support adherence, and identify needs requiring further care. |
| SUPPORT 2 | Caregiver/family support | Strengthen caregivers’ capacity to support patients’ recovery, prescribed exercises and aftercare, facilitate follow-up, and recognize warning signs. |
| SUPPORT 3 | Referral & Support | Identify recovery problems, warning signs, and unmet rehabilitation needs, and facilitate referral to appropriate care or rehabilitation services. |
| ENABLING | Training, supervision, tools and communication | Enable consistent, safe and coordinated delivery of the intervention within existing hospital and community health-system structures. |

**3. Package pathway**

| **01** | **HOSPITAL DISCHARGE**  Identify eligible patient and confirm discharge readiness. |
| --- | --- |

**▼**

| **02** | **STANDARDIZED DISCHARGE PREPARATION**  Provide recovery plan, mobility guidance, prescribed exercises, precautions, aftercare, danger signs and follow-up information. |
| --- | --- |

**▼**

| **03** | **PATIENT + CAREGIVER PREPARED**  Confirm understanding and provide simple, actionable recovery instructions. |
| --- | --- |

**▼**

| **04** | **HOSPITAL CHW/VHT/CHEW LINKAGE**  Link the patient to the appropriate community health worker before or immediately after discharge. |
| --- | --- |

**▼**

| **05** | **COMMUNITY / HOME FOLLOW-UP**  Assess mobility and barriers, reinforce the prescribed plan, support adherence, engage caregivers. |
| --- | --- |

**▼**

| **06** | **REFERRAL / SUPPORT**  Identify danger signs, deterioration or unmet rehabilitation needs and facilitate referral to the health facility. |
| --- | --- |

**▼**

| **07** | **FACILITY–COMMUNITY CONTINUITY**  Maintain communication between community and facility teams and support continued follow-up. |
| --- | --- |

**4. Package component 1: Standardized discharge preparation**

| **Element** | **Prototype specification** |
| --- | --- |
| Timing | Before discharge and reinforced at the point of discharge. |
| Provider | Responsible hospital clinical/orthopaedic/rehabilitation team member. |
| Recipient | Patient and caregiver/family member where available. |
| Fracture/recovery information | Simple explanation of recovery, precautions and expected follow-up. |
| Mobility plan | Mobility goals, precautions, prescribed home exercises and walking-aid guidance where applicable. |
| Aftercare | Medication, wound/aftercare instructions and danger signs. |
| Follow-up | Date, place and purpose of review. |
| Community linkage | CHW/VHT/CHEW contact/referral information and initiation of linkage. |
| Communication | Verbal explanation plus simple patient-facing written/visual information adapted to language and literacy. |
| Understanding check | Confirm that patient/caregiver understands key instructions before discharge. |

**5. Package component 2: Hospital-to-community linkage**

| **Step** | **Action** | **Responsible actor** | **Output** |
| --- | --- | --- | --- |
| 1 | Identify community location and eligibility for post-discharge follow-up. | Hospital team | Patient identified for linkage. |
| 2 | Obtain consent for community follow-up and record essential contact/location information. | Hospital team | Consent and linkage information recorded. |
| 3 | Initiate referral/linkage before or immediately after discharge. | Hospital team | Referral sent/communication initiated. |
| 4 | Receive referral and establish contact. | CHW/VHT/CHEW | Patient contacted/visit arranged. |
| 5 | Reinforce recovery plan and assess barriers. | CHW/VHT/CHEW | Follow-up information documented. |
| 6 | Identify danger signs, deterioration or unmet rehabilitation needs. | CHW/VHT/CHEW | Concern documented and escalation initiated where necessary. |
| 7 | Refer back to appropriate facility/service and communicate key information. | CHW/VHT/CHEW + facility | Referral/escalation completed. |

**6. Community/home follow-up package**

| **Follow-up function** | **Minimum activity** | **Safeguard** |
| --- | --- | --- |
| Assess | Ask about mobility progress, adherence, barriers, missed reviews and warning signs. | CHW/VHT/CHEW should not independently diagnose conditions or change the clinical treatment plan. |
| Reinforce | Reinforce the hospital-provided mobility/exercise and recovery plan. | CHW follows the prescribed plan rather than independently prescribing rehabilitation. |
| Support | Encourage safe participation, caregiver support and practical problem-solving. | CHW/VHT/CHEW adapts communication and support to the patient’s household and social context. |
| Identify | Recognize danger signs, deterioration or unmet rehabilitation needs. | CHW/VHT/CHEW uses agreed criteria to determine when referral is required. |
| Refer | Link patient back to the appropriate health facility/rehabilitation service. | CHW/VHT/CHEW follows the agreed referral pathway and communicates relevant information to the receiving service. |
| Document | Record contact, concerns, action and referral where applicable. | CHW/VHT/CHEW uses simple, standardized documentation tools.. |

**7. Caregiver/family support module**

- Explain the patient's mobility and recovery plan in simple language.
- Demonstrate how the caregiver can support safe mobility and prescribed exercises.
- Reinforce medication and wound/aftercare instructions as provided by the clinical team.
- Teach recognition of warning signs and when/where to seek help.
- Encourage practical support for attendance at scheduled reviews and community follow-up.

**8. Referral and escalation package**

| **Trigger category** | **Action** | **Destination** |
| --- | --- | --- |
| Danger signs / suspected complication | Prompt referral according to facility protocol. | Appropriate health facility/clinical service. |
| Deteriorating mobility or function | Communicate concern and arrange clinical/rehabilitation review. | Facility rehabilitation/clinical team. |
| Unmet rehabilitation need | Link to available rehabilitation service. | Physiotherapy/rehabilitation service. |
| Missed follow-up / loss of contact | Attempt locally feasible re-contact; escalate persistent concerns. | CHW supervisor/facility contact point. |
| Social/access barrier affecting recovery | Document barrier and link to available support/referral mechanism. | Relevant facility/community service. |

**9. Roles and accountability package**

| **Actor** | **Responsibilities** |
| --- | --- |
| Discharging hospital team | Identify patient; complete discharge preparation; confirm understanding; initiate community linkage; document referral. |
| Physiotherapy/rehabilitation staff | Provide or confirm clinically appropriate mobility/exercise plan; support referral decisions. |
| CHW/VHT/CHEW | Receive referral; contact/visit patient; reinforce plan; identify barriers/warning signs; facilitate referral; document follow-up. |
| Patient/caregiver | Participate in education; follow agreed recovery plan; attend review; report warning signs. |
| Facility supervisor | Monitor completion of discharge and linkage processes; support referral problem-solving and supervision. |
| District/programme manager | Coordinate implementation, supervision, reporting, training and resource planning. |

**10.** **Prototype tools included in the package**

| **Tool** | **Key fields / content** | **Primary user** |
| --- | --- | --- |
| Tool 1: Standardized discharge checklist | Patient identification; recovery information; mobility plan; exercises; walking aid; medication/aftercare; danger signs; follow-up; community linkage; understanding check. | Hospital team |
| Tool 2: Patient/caregiver recovery information sheet | Plain-language recovery instructions; mobility precautions; exercises as prescribed; warning signs; review details; community contact. | Patient/caregiver |
| Tool 3: Hospital-to-community referral/linkage form | Patient/location/contact; discharge date; key recovery instructions; follow-up needs; receiving CHW/VHT/CHEW; referral date. | Hospital + CHW/VHT/CHEW |
| Tool 4: Community follow-up checklist | Contact/visit date; mobility status; adherence/barriers; caregiver support; warning signs; action; referral. | CHW/VHT/CHEW |
| Tool 5: Referral/escalation record | Reason for referral; destination; date; urgency; action taken; feedback received. | CHW/VHT/CHEW + facility |
| Tool 6: Implementation monitoring dashboard | Eligible patients; discharge checklist completion; linkage; follow-up; referrals; fidelity; patient experience. | Facility/district team |

**11. Minimum implementation package**

| **Requirement** | **Minimum standard** |
| --- | --- |
| Training/orientation | Brief orientation for responsible hospital staff and CHWs/VHTs/CHEWs on roles, tools, communication, referral and safeguards. |
| SOP/workflow | Clear process showing who identifies, prepares, links, follows up and escalates. |
| Tools | Standardized discharge, linkage, follow-up and referral forms. |
| Referral network | Named facility/service contacts and agreed escalation process. |
| Supervision | Routine supportive supervision and review of implementation records. |
| Communication | Feasible mechanism for hospital–community contact and referral communication. |
| Adaptation | Local language/literacy and context adaptation while retaining minimum core functions. |
| Resources | Budget for orientation, materials, communication, supervision and feasible community follow-up. |

**12. Policy review and scale-up considerations**

The intervention package is designed to operate within **existing hospital and community health structures**, particularly routine discharge processes and CHW/VHT/CHEW systems, rather than creating a parallel service-delivery structure. Before wider implementation, the package should be reviewed for its **adoption, appropriateness, feasibility, fidelity, scalability, sustainability and resource requirements**.

| **Policy issue** | **Recommended review question** |
| --- | --- |
| Integration | Can the standardized discharge tool be embedded into routine orthopaedic/clinical discharge procedures? |
| Community workforce | Which existing community cadre is best positioned to deliver the linkage/follow-up function in each implementation setting? |
| Financing | What recurrent resources are required for training, tools, communication, supervision and community follow-up? |
| Referral | How can facility–community referral and feedback be incorporated into existing systems? |
| Equity | How will language, literacy, distance, transport and household support barriers be addressed? |
| Scale-up | What feasibility and effectiveness evidence is required before broader implementation? |

**13. Implementation monitoring framework**

| **Indicator** | **Definition / use** |
| --- | --- |
| Discharge preparation coverage | Eligible discharged patients receiving all minimum discharge components. |
| Linkage coverage | Eligible patients successfully linked to CHW/VHT/CHEW. |
| Community follow-up coverage | Linked patients receiving planned community contact. |
| Fidelity | Proportion of records meeting minimum intervention standards. |
| Referral completion | Proportion of indicated referrals successfully reaching the appropriate service. |
| Patient/caregiver experience | Perceived clarity, usefulness and acceptability of the package. |
| Continuity of care | Attendance/engagement with scheduled follow-up. |
| Mobility recovery | Study-defined mobility recovery outcome for subsequent evaluation. |

**14. Prototype status and next step**

**Status:** The intervention is currently a **stakeholder-refined intervention prototype package**, developed from the co-design process and the prioritized intervention components. The package now specifies the core and supporting intervention components, delivery pathway, roles and responsibilities, community follow-up activities, caregiver support, referral processes, implementation requirements and monitoring elements. However, it has **not yet undergone formal usability testing or field testing** and should not be presented as an established or effective intervention.

**Next steps:** Following this refinement, the intervention should undergo **feasibility/pilot evaluation** to assess its acceptability, appropriateness, feasibility, fidelity, reach and resource requirements before proceeding to actual implementation or wider scale-up.

**15. APPENDICES**

**APPENDIX I: STANDARDIZED DISCHARGE CHECKLIST**

A. Patient and Discharge Information

| **Item** | **Information** |
| --- | --- |
| Patient identification number | ____________________________ |
| Patient name/initials | ____________________________ |
| Age / Sex | ________________ / ________________ |
| Telephone/contact | ____________________________ |
| Caregiver name/contact | ____________________________ |
| Home/community location | ____________________________ |
| Date of admission / discharge | ________________ / ________________ |
| Discharging ward/unit | ____________________________ |
| Responsible provider | ____________________________ |
| Primary fracture/injury | ____________________________ |
| Planned follow-up date/place | ____________________________ |

# B. Standardized Discharge Preparation

| **Check** | **Minimum requirement** | **Comments** |
| --- | --- | --- |
| ☐ | Recovery and expected follow-up explained. | ____________ |
| ☐ | Mobility/activity plan explained. | ____________ |
| ☐ | Mobility precautions/restrictions explained. | ____________ |
| ☐ | Prescribed exercises/activities explained or demonstrated, where applicable. | ____________ |
| ☐ | Walking-aid use explained, where applicable. | ____________ |
| ☐ | Medication and aftercare instructions explained. | ____________ |
| ☐ | Wound/other relevant aftercare explained, where applicable. | ____________ |
| ☐ | Warning signs and when/where to seek care explained. | ____________ |
| ☐ | Scheduled review date, location and purpose explained. | ____________ |
| ☐ | Patient/caregiver information sheet provided, where applicable. | ____________ |

# C. Patient/Caregiver Understanding Check

| **Understanding area** | **Status** | **Clarification provided** |
| --- | --- | --- |
| Recovery/mobility plan | ☐ Yes ☐ No | ____________ |
| Mobility precautions | ☐ Yes ☐ No | ____________ |
| Prescribed exercises/activities | ☐ Yes ☐ No | ____________ |
| Walking-aid use, where applicable | ☐ Yes ☐ No ☐ N/A | ____________ |
| Medication/aftercare | ☐ Yes ☐ No | ____________ |
| Warning signs and where to seek help | ☐ Yes ☐ No | ____________ |
| Follow-up date/place | ☐ Yes ☐ No | ____________ |

# D. Caregiver and Family Support

| **Support activity** | **Status** | **Notes** |
| --- | --- | --- |
| Recovery plan explained to caregiver | ☐ Yes ☐ No ☐ N/A | ____________ |
| Safe mobility support explained/demonstrated | ☐ Yes ☐ No ☐ N/A | ____________ |
| Support for prescribed exercises/activities explained | ☐ Yes ☐ No ☐ N/A | ____________ |
| Medication/wound/aftercare instructions reinforced | ☐ Yes ☐ No ☐ N/A | ____________ |
| Warning signs and appropriate help explained | ☐ Yes ☐ No ☐ N/A | ____________ |
| Support for scheduled reviews/community follow-up discussed | ☐ Yes ☐ No ☐ N/A | ____________ |

# E. Hospital-to-Community Linkage

| **Linkage item** | **Details / status** |
| --- | --- |
| Community follow-up required | ☐ Yes ☐ No |
| CHW/VHT/CHEW identified | Name: __________________________ |
| CHW/VHT/CHEW contact | ____________________________ |
| Community location | ____________________________ |
| Referral/linkage initiated | ☐ Before discharge ☐ Immediately after discharge ☐ N/A |
| Key recovery information communicated | ☐ Yes ☐ No ☐ N/A |
| Expected first community contact | Date: __________________________ |
| Hospital contact for clarification/referral | ____________________________ |

# F. Referral and Follow-Up Needs

| **Need identified** | **Action / destination** | **Status** |
| --- | --- | --- |
| ☐ Physiotherapy/rehabilitation | ________________ | ☐ Completed ☐ Pending |
| ☐ Clinical/orthopaedic review | ________________ | ☐ Completed ☐ Pending |
| ☐ Other health service | ________________ | ☐ Completed ☐ Pending |
| ☐ Social/access support | ________________ | ☐ Completed ☐ Pending |
| ☐ No additional referral identified | N/A | ☐ |

# G. Final Discharge Verification

| **Verification** | **Status** |
| --- | --- |
| Minimum discharge information completed | ☐ Yes ☐ No |
| Patient/caregiver understanding checked | ☐ Yes ☐ No |
| Community linkage completed where required | ☐ Yes ☐ No ☐ N/A |
| Follow-up arrangements confirmed | ☐ Yes ☐ No |
| Outstanding concerns communicated to responsible provider | ☐ Yes ☐ No ☐ N/A |

# H. Sign-Off

| **Role** | **Name** | **Signature** | **Date** |
| --- | --- | --- | --- |
| Discharging provider | ____________ | ____________ | ________ |
| Physiotherapy/rehabilitation provider, if applicable | ____________ | ____________ | ________ |
| Patient | ____________ | ____________ | ________ |
| Caregiver, if applicable | ____________ | ____________ | ________ |

**APPENDIX II**: **PATIENT/CAREGIVER RECOVERY INFORMATION SHEET**

**Important:** This information sheet supports the recovery plan provided by your health-care team. Follow the specific instructions given to you by your doctor, physiotherapist or other qualified health-care provider.

**1. Your recovery plan**

After a lower limb fracture, recovery continues after leaving hospital. Your health-care team will provide a plan based on your injury and treatment. Keep this information sheet and use it together with the instructions given to you at discharge.

| **Your information** | **Details** |
| --- | --- |
| Patient name/ID | ________________________________________ |
| Date of discharge | ________________________________________ |
| Main injury/fracture | ________________________________________ |
| Mobility plan | ________________________________________ |
| Prescribed exercises/activities | ________________________________________ |
| Walking aid, if applicable | ________________________________________ |
| Next review date | ________________________________________ |
| Review facility/service | ________________________________________ |
| CHW/VHT/CHEW name/contact | ________________________________________ |
| Hospital contact/service | ________________________________________ |

**2. Mobility and safe activity**

Your mobility plan should follow the instructions provided by your clinical or rehabilitation team.

Follow the mobility instructions given to you at discharge.

Use your prescribed walking aid, if applicable, according to the instructions provided by the health-care team.

Carry out only the exercises or activities that have been prescribed or approved for you.

Ask your health-care provider or CHW/VHT/CHEW for clarification if you are unsure about an activity.

Avoid changing your mobility or exercise plan on your own.

**3. Prescribed exercises and recovery activities**

Your health-care provider may prescribe exercises or activities to support your recovery. Use the space below to record the instructions given to you.

| **Exercise/activity** | **How often / instructions** | **Notes** |
| --- | --- | --- |
| 1. ____________________ | ________________________ | ________________ |
| 2. ____________________ | ________________________ | ________________ |
| 3. ____________________ | ________________________ | ________________ |

**4. Medication and aftercare**

Take medicines according to the instructions provided by your clinical team.

Follow the wound-care and other aftercare instructions provided at discharge, where applicable.

Do not change prescribed treatment without discussing it with an appropriate health-care provider.

If you have questions or problems with your medicines or aftercare, contact the appropriate health service.

**5. Warning signs: when to seek help**

**Seek prompt medical advice if you notice a warning sign or if your condition is getting worse.** The specific warning signs relevant to your injury and treatment should be explained by your clinical team before discharge.

Examples of concerns that should be discussed promptly with the health-care team include:

New or worsening problems with mobility or function.

Unexpected worsening of pain or other symptoms.

Problems with the wound or other concerns identified by the clinical team.

Any warning sign specifically explained to you by your doctor or rehabilitation provider.

Your health-care team should complete the specific warning signs for your condition here:

| **Warning sign / concern** | **What to do / where to seek help** |
| --- | --- |
| ________________________________ | ________________________________ |
| ________________________________ | ________________________________ |
| ________________________________ | ________________________________ |

**6. Follow-up appointments**

Attend the follow-up appointments arranged by your health-care team. These visits help the team review your recovery and provide further care when needed.

| **Date** | **Facility/service** | **Purpose** | **Attended** |
| --- | --- | --- | --- |
| ____________ | ________________ | ________________ | ☐ Yes ☐ No |
| ____________ | ________________ | ________________ | ☐ Yes ☐ No |
| ____________ | ________________ | ________________ | ☐ Yes ☐ No |

**7. Community/home follow-up**

Where community follow-up is arranged, a CHW/VHT/CHEW may contact or visit you at home. The community health worker will help reinforce the recovery plan provided by the health facility, ask about your progress and barriers, support adherence, identify concerns and facilitate referral when further care is needed.

Tell the CHW/VHT/CHEW about difficulties following your recovery plan.

Tell the CHW/VHT/CHEW about missed reviews or problems affecting your recovery.

Ask questions if you do not understand the instructions provided by the health facility.

The CHW/VHT/CHEW does not replace your clinical or rehabilitation provider and should not independently change your treatment or prescribe new rehabilitation activities.

**8. How caregivers and family members can help**

Support the patient to follow the prescribed recovery plan.

Provide safe assistance with mobility and prescribed exercises where instructed.

Support medication, wound care and other aftercare as directed by the clinical team.

Help the patient attend scheduled hospital/rehabilitation reviews and community follow-up.

Watch for warning signs and communicate concerns promptly.

Encourage the patient and provide practical support within the household.

**9. My questions and concerns**

| **Question/concern** | **Action or response** |
| --- | --- |
| ________________________________________ | ________________________________________ |
| ________________________________________ | ________________________________________ |
| ________________________________________ | ________________________________________ |

**10. Important contacts**

| **Contact** | **Name/service** | **Telephone** |
| --- | --- | --- |
| Hospital/clinical team | ________________________ | ________________ |
| Physiotherapy/rehabilitation | ________________________ | ________________ |
| CHW/VHT/CHEW | ________________________ | ________________ |
| Other agreed contact | ________________________ | ________________ |
| **Remember:** Keep your recovery information sheet and discharge instructions. Follow the plan provided by your health-care team, attend scheduled reviews, and seek help when you notice a concern or warning sign. | | |

**APPENDIX III: HOSPITAL-TO-COMMUNITY LINKAGE FORM**

**Purpose:** To establish a clear and documented link between the hospital and the patient's home/community through an existing CHW/VHT/CHEW structure following discharge.

Use this form when a patient is identified for community follow-up. The hospital team completes the referral/linkage information and communicates the relevant recovery plan to the receiving CHW/VHT/CHEW. The community worker completes the receipt and follow-up sections.

**A. Patient and Discharge Information**

| **Item** | **Details** |
| --- | --- |
| Patient identification number | ________________________________________ |
| Patient name/initials | ________________________________________ |
| Age / Sex | ________________ / ________________ |
| Telephone/contact | ________________________________________ |
| Caregiver name/contact | ________________________________________ |
| Home/community location | ________________________________________ |
| Village/area | ________________________________________ |
| Date of discharge | ________________________________________ |
| Discharging ward/unit | ________________________________________ |
| Hospital | ________________________________________ |

**B. Reason for Community Linkage**

| **Linkage item** | **Status / details** |
| --- | --- |
| Eligible for community follow-up | ☐ Yes ☐ No |
| Patient/caregiver consent for community follow-up | ☐ Yes ☐ No |
| Mobility/recovery support required | ☐ Yes ☐ No |
| Caregiver/family support required | ☐ Yes ☐ No |
| Follow-up/review support required | ☐ Yes ☐ No |
| Other identified need | ________________________________________ |

**C. Recovery and Follow-Up Information to be Communicated**

| **Information** | **Details** |
| --- | --- |
| Mobility/activity plan | ________________________________________ |
| Mobility precautions/restrictions | ________________________________________ |
| Prescribed exercises/activities | ________________________________________ |
| Walking aid, where applicable | ________________________________________ |
| Medication/aftercare instructions | ________________________________________ |
| Warning signs explained to patient/caregiver | ________________________________________ |
| Scheduled review date | ________________________________________ |
| Review facility/service | ________________________________________ |
| Other relevant instructions | ________________________________________ |

**D. Receiving CHW/VHT/CHEW Details**

| **Item** | **Details** |
| --- | --- |
| Community health worker cadre | ☐ CHW ☐ VHT ☐ CHEW |
| Name | ________________________________________ |
| Telephone/contact | ________________________________________ |
| Community/area served | ________________________________________ |
| Supervisor/contact, if applicable | ________________________________________ |
| Date referral/linkage initiated | ________________________________________ |
| Method of communication | ☐ Phone ☐ Written form ☐ In person ☐ Other: __________ |

**E. Hospital Referral and Handover**

| **Handover activity** | **Status** |
| --- | --- |
| Patient location/contact information communicated | ☐ Yes ☐ No |
| Recovery/mobility plan communicated | ☐ Yes ☐ No |
| Prescribed exercises/activities communicated | ☐ Yes ☐ No ☐ N/A |
| Follow-up appointment details communicated | ☐ Yes ☐ No |
| Warning signs communicated | ☐ Yes ☐ No |
| Caregiver/support needs communicated | ☐ Yes ☐ No ☐ N/A |
| Relevant clinical/rehabilitation information communicated | ☐ Yes ☐ No ☐ N/A |

**F. CHW/VHT/CHEW Receipt and Initial Contact**

| **Item** | **Details / status** |
| --- | --- |
| Referral received | ☐ Yes ☐ No |
| Date referral received | ________________________________________ |
| Patient/caregiver contacted | ☐ Yes ☐ No |
| Date of first contact | ________________________________________ |
| Contact method | ☐ Home visit ☐ Phone ☐ Other: __________ |
| Patient/caregiver available | ☐ Yes ☐ No |
| Initial concerns identified | ________________________________________ |
| Barriers to recovery identified | ________________________________________ |
| Further care/referral needed | ☐ Yes ☐ No |

**G. Initial Community Follow-Up Plan**

| **Follow-up activity** | **Plan / details** |
| --- | --- |
| First community follow-up | Date: ______________________________ |
| Main purpose of follow-up | ________________________________________ |
| Mobility/recovery areas to reinforce | ________________________________________ |
| Caregiver support required | ________________________________________ |
| Potential barriers to address | ________________________________________ |
| Follow-up/review attendance support | ________________________________________ |
| Referral/further care anticipated | ☐ Yes ☐ No ☐ To be determined |
| Next planned contact | Date: ______________________________ |

**H. Referral and Further Care**

| **Concern/need identified** | **Action / destination** | **Date / status** |
| --- | --- | --- |
| ☐ Mobility deterioration | ____________________________ | ________________ |
| ☐ Warning sign/clinical concern | ____________________________ | ________________ |
| ☐ Unmet rehabilitation need | ____________________________ | ________________ |
| ☐ Missed follow-up/loss of contact | ____________________________ | ________________ |
| ☐ Social/access barrier | ____________________________ | ________________ |
| ☐ Other | ____________________________ | ________________ |

**I. Communication and Feedback**

| **Item** | **Details** |
| --- | --- |
| Concern communicated to hospital/facility | ☐ Yes ☐ No ☐ N/A |
| Receiving service/facility | ________________________________________ |
| Date communicated | ________________________________________ |
| Person contacted | ________________________________________ |
| Feedback received | ☐ Yes ☐ No ☐ Pending |
| Action/outcome | ________________________________________ |
| **Role boundary and safety:** The CHW/VHT/CHEW reinforces the recovery plan provided by the health facility, identifies barriers and concerns, supports follow-up and facilitates referral. The CHW/VHT/CHEW should not independently diagnose, change clinical treatment, or prescribe or modify rehabilitation activities. | |

**J. Sign-Off and Handover Confirmation**

| **Role** | **Name** | **Signature** | **Date** |
| --- | --- | --- | --- |
| Hospital/discharging provider | ________________ | ________________ | ____________ |
| Physiotherapy/rehabilitation provider, if applicable | ________________ | ________________ | ____________ |
| Receiving CHW/VHT/CHEW | ________________ | ________________ | ____________ |
| Patient/caregiver, where applicable | ________________ | ________________ | ____________ |

**Minimum linkage standard**

For an eligible patient requiring community follow-up, linkage should include: identification of the receiving CHW/VHT/CHEW; consent for community follow-up; essential patient/location/contact information; communication of the relevant recovery and follow-up plan; confirmation that the referral was received; and documentation of the initial community contact.

**APPENDIX IV**: **CHW/VHT/CHEW COMMUNITY FOLLOW-UP CHECKLIST**

**Purpose:** To guide CHWs/VHTs/CHEWs in providing structured community/home follow-up after discharge, reinforcing the facility-provided recovery plan, identifying barriers and concerns, supporting adherence, and facilitating referral when further care is needed.

Use this checklist at each planned community contact. The CHW/VHT/CHEW should follow the recovery plan provided by the health facility and should not independently diagnose, change clinical treatment, or prescribe or modify rehabilitation activities.

A. Patient and Contact Information

| **Item** | **Details** |
| --- | --- |
| Patient identification number | ________________________________________ |
| Patient name/initials | ________________________________________ |
| Age / Sex | ________________ / ________________ |
| Telephone/contact | ________________________________________ |
| Caregiver name/contact | ________________________________________ |
| Community/location | ________________________________________ |
| Date of discharge | ________________________________________ |
| Date of current contact | ________________________________________ |
| CHW/VHT/CHEW name | ________________________________________ |
| Contact number | ________________________________________ |
| Type of contact | ☐ Home visit ☐ Phone ☐ Other: __________ |
| Contact number since discharge | ☐ First contact ☐ 2nd ☐ 3rd ☐ Other: ______ |

# B. Contact and Patient Status

| **Item** | **Status / notes** |
| --- | --- |
| Patient reached | ☐ Yes ☐ No |
| Caregiver available, where applicable | ☐ Yes ☐ No ☐ N/A |
| Patient reports following the recovery plan | ☐ Yes ☐ Partly ☐ No |
| Patient has attended scheduled review | ☐ Yes ☐ No ☐ Not yet due |
| New concern reported | ☐ Yes ☐ No |
| General progress since last contact | ________________________________________ |

# C. Assess: Mobility and Recovery Progress

| **Assessment area** | **What to ask/check** | **Finding / notes** |
| --- | --- | --- |
| Mobility progress | How is your ability to move/walk compared with the previous contact? | ________________ |
| Daily activities | Are you having difficulty with usual activities because of the injury? | ________________ |
| Prescribed plan | Are you able to follow the mobility/recovery instructions provided by the facility? | ________________ |
| Exercises/activities | Are you able to carry out the prescribed exercises/activities? | ________________ |
| Walking aid | Are you using the prescribed walking aid where applicable? | ________________ |
| Follow-up attendance | Have you attended the scheduled hospital/rehabilitation review? | ________________ |

# D. Identify: Barriers and Concerns

| **Potential barrier/concern** | **Identified?** | **Details / action needed** |
| --- | --- | --- |
| Pain or other difficulty affecting recovery | ☐ Yes ☐ No | ____________________________ |
| Difficulty following prescribed exercises/activities | ☐ Yes ☐ No | ____________________________ |
| Difficulty using walking aid | ☐ Yes ☐ No ☐ N/A | ____________________________ |
| Physical/home environment barrier | ☐ Yes ☐ No | ____________________________ |
| Transport/access barrier | ☐ Yes ☐ No | ____________________________ |
| Financial/resource barrier | ☐ Yes ☐ No | ____________________________ |
| Limited caregiver/family support | ☐ Yes ☐ No | ____________________________ |
| Missed appointment/follow-up | ☐ Yes ☐ No | ____________________________ |
| Other concern | ☐ Yes ☐ No | ____________________________ |

# E. Reinforce: Recovery Plan

| **Activity** | **Completed** | **Notes** |
| --- | --- | --- |
| Recovery/mobility plan reinforced | ☐ Yes ☐ No | ____________________________ |
| Mobility precautions reviewed | ☐ Yes ☐ No | ____________________________ |
| Prescribed exercises/activities reviewed | ☐ Yes ☐ No ☐ N/A | ____________________________ |
| Walking-aid guidance reinforced, where applicable | ☐ Yes ☐ No ☐ N/A | ____________________________ |
| Medication/aftercare instructions reinforced as provided by clinical team | ☐ Yes ☐ No ☐ N/A | ____________________________ |
| Follow-up appointment information reinforced | ☐ Yes ☐ No | ____________________________ |

# F. Support: Patient and Caregiver

| **Support activity** | **Status** | **Notes** |
| --- | --- | --- |
| Patient encouraged to continue the agreed recovery plan | ☐ Yes ☐ No | ____________________________ |
| Caregiver support discussed/provided, where applicable | ☐ Yes ☐ No ☐ N/A | ____________________________ |
| Safe mobility support discussed | ☐ Yes ☐ No ☐ N/A | ____________________________ |
| Support for prescribed exercises discussed | ☐ Yes ☐ No ☐ N/A | ____________________________ |
| Practical barriers discussed and problem-solving supported | ☐ Yes ☐ No | ____________________________ |
| Attendance at follow-up supported | ☐ Yes ☐ No ☐ N/A | ____________________________ |

# G. Identify Warning Signs or Need for Further Care

| **Concern** | **Present?** | **Action** |
| --- | --- | --- |
| Warning sign/clinical concern identified | ☐ Yes ☐ No | ____________________________ |
| Deterioration in mobility/function | ☐ Yes ☐ No | ____________________________ |
| Unmet rehabilitation need | ☐ Yes ☐ No | ____________________________ |
| Missed follow-up/loss of contact | ☐ Yes ☐ No | ____________________________ |
| Social/access problem affecting recovery | ☐ Yes ☐ No | ____________________________ |
| Other concern requiring further care | ☐ Yes ☐ No | ____________________________ |

# H. Referral and Follow-Up Action

| **Action** | **Details** |
| --- | --- |
| No further action required | ☐ Yes ☐ No |
| Reinforcement/support only | ☐ Yes ☐ No |
| Referral to clinical service | Destination: ______________________________ |
| Referral to physiotherapy/rehabilitation | Destination: ______________________________ |
| Contact facility/supervisor for guidance | Name/service: ______________________________ |
| Urgency according to agreed pathway | ☐ Routine ☐ Prompt ☐ Urgent |
| Referral date | ____________________________ |
| Next follow-up date | ____________________________ |

# I. Referral Outcome / Feedback

| **Item** | **Status / details** |
| --- | --- |
| Patient reached referred service | ☐ Yes ☐ No ☐ Pending |
| Service/facility contacted | ____________________________ |
| Date contacted/attended | ____________________________ |
| Feedback received | ☐ Yes ☐ No ☐ Pending |
| Outcome/action taken | ____________________________ |
| Additional follow-up required | ☐ Yes ☐ No |

# J. Documentation and Next Steps

| **Documentation item** | **Status** |
| --- | --- |
| Current contact documented | ☐ Yes ☐ No |
| Concerns/barriers documented | ☐ Yes ☐ No ☐ N/A |
| Referral documented, where applicable | ☐ Yes ☐ No ☐ N/A |
| Patient/caregiver informed of next step | ☐ Yes ☐ No |
| Next community contact agreed | ☐ Yes ☐ No |
| Next contact date | ____________________________ |
| **Role boundary and safety:** CHWs/VHTs/CHEWs reinforce the recovery plan provided by the health facility, support adherence, identify barriers and concerns, and facilitate referral. They should not independently diagnose complications, change clinical treatment, or prescribe or modify rehabilitation activities. | |

# Minimum follow-up standard

At each planned community contact, the CHW/VHT/CHEW should document patient contact, mobility/recovery progress, adherence, barriers, caregiver support, warning signs or other concerns, actions taken, referrals where applicable, and the next follow-up step.

**APPENDIX V: REFERRAL AND FOLLOW-UP FORM**

**Purpose:** To document concerns identified during post-discharge follow-up, facilitate referral to appropriate clinical or rehabilitation services, and record referral completion and follow-up.

Use this form when a CHW/VHT/CHEW or other responsible team member identifies a problem, warning sign, deterioration in mobility/function, unmet rehabilitation need, missed follow-up, or another concern requiring further care.

A. Patient and Referral Information

| **Item** | **Details** |
| --- | --- |
| Patient identification number | ________________________________________ |
| Patient name/initials | ________________________________________ |
| Age / Sex | ________________ / ________________ |
| Telephone/contact | ________________________________________ |
| Caregiver name/contact | ________________________________________ |
| Community/location | ________________________________________ |
| Date of discharge | ________________________________________ |
| Date concern identified | ________________________________________ |
| Person initiating referral | ________________________________________ |
| Cadre | ☐ CHW ☐ VHT ☐ CHEW ☐ Other: __________ |

# B. Reason for Referral

| **Reason / concern** | **Present?** | **Details** |
| --- | --- | --- |
| Warning sign / clinical concern | ☐ Yes ☐ No | ________________________________ |
| Deterioration in mobility or function | ☐ Yes ☐ No | ________________________________ |
| Unmet rehabilitation need | ☐ Yes ☐ No | ________________________________ |
| Difficulty following prescribed recovery plan | ☐ Yes ☐ No | ________________________________ |
| Missed follow-up / loss of contact | ☐ Yes ☐ No | ________________________________ |
| Social, transport or access barrier affecting recovery | ☐ Yes ☐ No | ________________________________ |
| Other concern | ☐ Yes ☐ No | ________________________________ |

# C. Concern and Relevant Information

| **Information to communicate** | **Details** |
| --- | --- |
| Patient's reported concern | ________________________________________ |
| Observed/reported change in mobility or function | ________________________________________ |
| Relevant barrier affecting recovery | ________________________________________ |
| Relevant prescribed recovery plan | ________________________________________ |
| Follow-up appointment due/missed | ________________________________________ |
| Other relevant information | ________________________________________ |

# D. Referral Decision and Destination

| **Item** | **Details / status** |
| --- | --- |
| Referral required | ☐ Yes ☐ No |
| Type of further care | ☐ Clinical ☐ Physiotherapy/rehabilitation ☐ Other: __________ |
| Receiving facility/service | ________________________________________ |
| Receiving provider/contact | ________________________________________ |
| Referral date | ________________________________________ |
| Agreed urgency | ☐ Routine ☐ Prompt ☐ Urgent — according to agreed pathway |
| Mode of referral | ☐ Patient directed ☐ Accompanied ☐ Phone/communication ☐ Written referral ☐ Other |

# E. Referral Handover

| **Handover information** | **Status** |
| --- | --- |
| Patient identification/contact information provided | ☐ Yes ☐ No |
| Reason for referral communicated | ☐ Yes ☐ No |
| Relevant recovery/mobility information communicated | ☐ Yes ☐ No |
| Relevant follow-up information communicated | ☐ Yes ☐ No |
| Caregiver information/support needs communicated | ☐ Yes ☐ No ☐ N/A |
| Receiving service acknowledged referral | ☐ Yes ☐ No ☐ Pending |

# F. Patient/Family Follow-Up After Referral

| **Follow-up item** | **Status / details** |
| --- | --- |
| Patient/caregiver informed about referral | ☐ Yes ☐ No |
| Patient attended/reached referred service | ☐ Yes ☐ No ☐ Pending |
| Date attended/reached | ________________________________________ |
| Care received / action taken | ________________________________________ |
| Further appointment required | ☐ Yes ☐ No ☐ Unknown |
| Next appointment/date | ________________________________________ |
| Additional support required | ________________________________________ |

# G. Referral Outcome and Feedback

| **Item** | **Details** |
| --- | --- |
| Referral outcome | ☐ Completed ☐ Pending ☐ Not completed |
| Outcome/feedback received from service | ________________________________________ |
| Further clinical/rehabilitation follow-up required | ☐ Yes ☐ No |
| Further community follow-up required | ☐ Yes ☐ No |
| Additional referral required | ☐ Yes ☐ No |
| Action taken following feedback | ________________________________________ |
| Date outcome recorded | ________________________________________ |

# H. Follow-Up Plan

| **Next step** | **Details** |
| --- | --- |
| Next CHW/VHT/CHEW contact | Date: ______________________________ |
| Purpose of next contact | ________________________________________ |
| Follow-up responsibility | ________________________________________ |
| Facility/service follow-up | ________________________________________ |
| Patient/caregiver action required | ________________________________________ |
| Other agreed action | ________________________________________ |

# I. Communication and Documentation

| **Documentation item** | **Status** |
| --- | --- |
| Referral recorded in community follow-up record | ☐ Yes ☐ No |
| Receiving facility/service documented | ☐ Yes ☐ No |
| Referral outcome documented | ☐ Yes ☐ No ☐ Pending |
| Patient/caregiver informed of next step | ☐ Yes ☐ No |
| Feedback communicated to relevant team | ☐ Yes ☐ No ☐ N/A |
| Form filed/recorded according to local procedure | ☐ Yes ☐ No |
| **Role boundary and safety:** The CHW/VHT/CHEW identifies concerns, reinforces the facility-provided recovery plan and facilitates referral. The CHW/VHT/CHEW should not independently diagnose, change clinical treatment, prescribe medication, or prescribe or modify rehabilitation activities. | |

# J. Sign-Off

| **Role** | **Name** | **Signature** | **Date** |
| --- | --- | --- | --- |
| CHW/VHT/CHEW initiating referral | ________________ | ________________ | ____________ |
| Receiving facility/service, where applicable | ________________ | ________________ | ____________ |
| Patient/caregiver, where applicable | ________________ | ________________ | ____________ |

# Minimum referral and follow-up standard

When a concern requiring further care is identified, the referral should document the reason for referral, appropriate destination, relevant information communicated, action taken, referral status and subsequent follow-up. Where feasible, the outcome or feedback from the receiving service should be recorded and communicated to the relevant community/facility team.

**APPENDIX VI: IMPLEMENTATION MONITORING DASHBOARD**

**Purpose:** To provide a simple routine monitoring tool for assessing intervention reach, coverage, fidelity, referral completion, patient/caregiver experience and continuity of care.

**A. Monitoring period and site information**

| **Item** | **Details** |
| --- | --- |
| Reporting period | From: ____________ To: ____________ |
| Facility / site | ________________________________________ |
| District / area | ________________________________________ |
| Reporting team / supervisor | ________________________________________ |
| Date completed | ________________________________________ |

**B. Core Implementation Indicators**

| **Indicator** | **Definition / numerator and denominator** | **Data source** | **Target*** | **Actual** | **Coverage / %** | **Status / action** |
| --- | --- | --- | --- | --- | --- | --- |
| Eligible patients | Number of patients eligible for the intervention during the reporting period. | Patient/discharge records | — | ____ | — | ____________ |
| Discharge preparation coverage | Eligible patients receiving all minimum discharge components ÷ eligible discharged patients ×100. | Discharge checklist | ____% | ____ | ____% | ____________ |
| Understanding-check completion | Eligible patients with documented patient/caregiver understanding check ÷ eligible discharged patients ×100. | Discharge checklist | ____% | ____ | ____% | ____________ |
| Community linkage coverage | Eligible patients successfully linked to CHW/VHT/CHEW ÷ eligible patients requiring community follow-up ×100. | Linkage form | ____% | ____ | ____% | ____________ |
| Community follow-up coverage | Linked patients receiving planned community contact ÷ patients successfully linked ×100. | CHW/VHT/CHEW follow-up checklist | ____% | ____ | ____% | ____________ |
| Referral initiation | Patients with an identified need for referral for whom referral was initiated ÷ patients identified as needing referral ×100. | Referral/follow-up form | ____% | ____ | ____% | ____________ |
| Referral completion | Indicated referrals completed ÷ indicated referrals initiated ×100. | Referral/follow-up form / facility records | ____% | ____ | ____% | ____________ |
| Continuity of care | Patients attending/engaging with scheduled follow-up ÷ patients due for follow-up ×100. | Follow-up records | ____% | ____ | ____% | ____________ |

* Targets should be finalized during technical review/feasibility testing and should not be interpreted as established effectiveness thresholds.

**C. Intervention Fidelity Monitoring**

| **Fidelity domain** | **Minimum standard to check** | **Records reviewed** | **Met standard** | **Gap identified / action** |
| --- | --- | --- | --- | --- |
| Discharge preparation | Minimum recovery, mobility, aftercare, warning-sign and follow-up information documented. | ____ | ☐ Yes ☐ No | ________________ |
| Understanding check | Patient/caregiver understanding check documented. | ____ | ☐ Yes ☐ No | ________________ |
| Community linkage | Eligible patients requiring linkage have documented CHW/VHT/CHEW referral/linkage. | ____ | ☐ Yes ☐ No | ________________ |
| Community follow-up | CHW/VHT/CHEW contact includes assessment, reinforcement, support, identification/referral and documentation as applicable. | ____ | ☐ Yes ☐ No | ________________ |
| Caregiver support | Caregiver involvement documented where applicable. | ____ | ☐ Yes ☐ No ☐ N/A | ________________ |
| Referral/follow-up | Identified concerns have documented referral/action and follow-up where required. | ____ | ☐ Yes ☐ No ☐ N/A | ________________ |
| Documentation | Required tools are complete and legible. | ____ | ☐ Yes ☐ No | ________________ |

**D. Community Follow-Up and Referral Monitoring**

| **Measure** | **Number** | **Notes** |
| --- | --- | --- |
| Patients due for community follow-up | ____ | ________________ |
| Patients contacted | ____ | ________________ |
| Patients not reached | ____ | ________________ |
| Patients with barriers to recovery identified | ____ | ________________ |
| Patients with warning signs/clinical concerns identified | ____ | ________________ |
| Patients with deteriorating mobility/function identified | ____ | ________________ |
| Patients with unmet rehabilitation needs identified | ____ | ________________ |
| Referrals initiated | ____ | ________________ |
| Referrals completed | ____ | ________________ |
| Referrals pending | ____ | ________________ |
| Patients requiring additional community follow-up | ____ | ________________ |

**E. Patient/Caregiver Experience**

| **Experience indicator** | **Patients/caregivers assessed** | **Positive / satisfactory** | **Concerns identified** | **Action** |
| --- | --- | --- | --- | --- |
| Clarity of discharge information | ____ | ____ | ____ | ________________ |
| Understanding of recovery/mobility plan | ____ | ____ | ____ | ________________ |
| Usefulness of patient information sheet | ____ | ____ | ____ | ________________ |
| Acceptability of CHW/VHT/CHEW follow-up | ____ | ____ | ____ | ________________ |
| Perceived usefulness of community support | ____ | ____ | ____ | ________________ |
| Ease of accessing follow-up/referral services | ____ | ____ | ____ | ________________ |

**F. Implementation Barriers and Corrective Actions**

| **Barrier / implementation problem** | **Frequency / extent** | **Likely cause** | **Action required** | **Responsible person** | **Due date** |
| --- | --- | --- | --- | --- | --- |
| ____________________________ | ________ | ________________ | ________________ | ________________ | ________ |
| ____________________________ | ________ | ________________ | ________________ | ________________ | ________ |
| ____________________________ | ________ | ________________ | ________________ | ________________ | ________ |
| ____________________________ | ________ | ________________ | ________________ | ________________ | ________ |

**G. Supervision and Quality Improvement**

| **Supervision item** | **Status / finding** | **Action** |
| --- | --- | --- |
| Routine record review completed | ☐ Yes ☐ No | ________________ |
| Discharge checklist completeness reviewed | ☐ Yes ☐ No | ________________ |
| Linkage records reviewed | ☐ Yes ☐ No | ________________ |
| Community follow-up records reviewed | ☐ Yes ☐ No | ________________ |
| Referral records reviewed | ☐ Yes ☐ No ☐ N/A | ________________ |
| CHW/VHT/CHEW support needs identified | ☐ Yes ☐ No | ________________ |
| Additional training/orientation needed | ☐ Yes ☐ No | ________________ |
| Workflow or referral pathway problem identified | ☐ Yes ☐ No | ________________ |

**H. Summary for the Reporting Period**

| **Summary area** | **Key finding / action** |
| --- | --- |
| Overall implementation performance | ____________________________________________ |
| Major achievement | ____________________________________________ |
| Main implementation gap | ____________________________________________ |
| Priority action for next period | ____________________________________________ |
| Support required from facility/district team | ____________________________________________ |

**I. Sign-Off**

| **Role** | **Name** | **Signature** | **Date** |
| --- | --- | --- | --- |
| CHW/VHT/CHEW supervisor | ________________ | ________________ | ____________ |
| Facility/programme focal person | ________________ | ________________ | ____________ |
| District/programme manager, where applicable | ________________ | ________________ | ____________ |
| **Interpretation and use:** This dashboard is intended for implementation monitoring, supportive supervision and quality improvement. It is not an effectiveness outcome measure. Indicators and targets should be finalized during technical review and feasibility testing. | | | |

The dashboard follows the intervention prototype's proposed monitoring domains: discharge preparation coverage, community linkage coverage, community follow-up coverage, fidelity, referral completion, patient/caregiver experience, continuity of care and the subsequent mobility recovery outcome. Mobility recovery should be measured separately using the study's defined outcome measure.
