# Supplementary material for: Co-designing a post-discharge intervention to improve mobility recovery among patients with lower limb fractures using human-centered design in greater Kampala, Uganda
Source: PLoS One. 2026 Nov 10;21(11):e0360232. doi: 10.1371/journal.pone.0360232 (PMC13750591; doi:10.1371/journal.pone.0360232)
Supplement: Supplementary file 2 [file pone.0360232.s004.docx]

**PARTICIPANT-INFORMED INTERVENTION PRIORITIZATION MATRIX**

*Co-designing a Post-Discharge Intervention to Improve Mobility Recovery among Patients with Lower Limb Fractures in Greater Kampala, Uganda*

**Purpose.** This matrix summarizes intervention components discussed by participants during co-design group discussions with Community Health Workers (CHWs/VHTs) and healthcare providers, including the implementation considerations that informed prioritization. The discussions resulted in two core prioritized interventions: **hospital-to-community linkage through CHEWs/VHTs** and **standardized discharge preparation tools**.

| **Proposed intervention component** | **Acceptability** | **Adoption** | **Appropriateness** | **Feasibility** | **Fidelity** | **Scalability** | **Compatibility** | **Sustainability** | **Costs** | **Penetration** | **Prioritization** |
| --- | --- | --- | --- | --- | --- | --- | --- | --- | --- | --- | --- |
| **Strengthened discharge preparation** | High | High | High | High | High | High | High | High | Low–Moderate | High | **PRIORITY 1** |
| **Caregiver engagement at discharge** | High | Moderate–High | High | Moderate | High | High | Moderate | High | Low | High | Supporting / enabling |
| **Hospital-to-community linkage system** | High | High | High | High | High | High | High | High | Moderate | High | **PRIORITY 2** |
| **Community-based and home follow-up support** | High | High | High | Moderate–High | Moderate–High | Moderate–High | High | Moderate–High | Moderate | High | Supporting / enabling |
| **Health worker follow-up support** | High | High | High | Moderate | Moderate | High | High | Moderate–High | Moderate | High | Supporting / enabling |
| **VHT/CHW training and role preparation** | High | High | High | Moderate | High | High | High | High | Moderate | High | Supporting / enabling |
| **Community sensitization and local stakeholder engagement** | High | Moderate–High | High | Moderate | Moderate | High | High | Moderate–High | Moderate | Moderate–High | Supporting / enabling |
| **Engagement of traditional bone setters** | Moderate–High | Moderate | High | Moderate | Moderate | Moderate | Moderate–High | Moderate | Moderate | Moderate–High | Supporting / enabling |
| **Decentralized follow-up and rehabilitation support** | High | Moderate–High | High | Moderate | Moderate–High | Moderate–High | High | Moderate | Moderate–High | High | Supporting / enabling |
| **Patients and family's recovery support** | High | High | High | Moderate | High | High | Moderate | High | Low–Moderate | High | Supporting / enabling |

**Interpretation of ratings:** High = strong fit for the intervention package based on the co-design priorities and implementation context; Moderate–High = favorable but dependent on supporting structures; Moderate = potentially useful but requiring additional resources, coordination or refinement; Low–Moderate = relatively less resource-intensive or potentially less demanding where applicable.
