# Supplementary material for: Co-designing a post-discharge intervention to improve mobility recovery among patients with lower limb fractures using human-centered design in greater Kampala, Uganda
Source: PLoS One. 2026 Nov 10;21(11):e0360232. doi: 10.1371/journal.pone.0360232 (PMC13750591; doi:10.1371/journal.pone.0360232)
Supplement: Supplementary file 3 [file pone.0360232.s005.docx]

**CODEBOOK**

Co-Designing a Post-Discharge Intervention to improve mobility recovery for patients with lower limb bone fractures *using Human-Centered Design in Uganda*

| **Theme** | **Sub-theme** | **Codes** | **Definition** |
| --- | --- | --- | --- |
| **Theme 1. Post-discharge care discontinuity** | **Absence of Structured Follow-Up** | • no follow-up system  • no support after discharge • no routine patient monitoring • no organised post-discharge review | Code: Any mention of the lack of a formal or organised system for monitoring, reviewing, or supporting patients after discharge. |
|  | **Delayed or Missed Review** | • missed review appointment • late return to hospital • did not attend review | Code: Any mention of a patient returning late for review, missing a scheduled appointment, or failing to attend follow-up altogether. |
|  | **Transport Barriers** | • transport cost • no money for transport • distance to health facility | Code: Any mention of financial, logistical, or transport-related difficulties that make it difficult to return to a health facility for review or care. |
|  | **Physical Mobility Constraints** | • unable to walk to facility • pain limits travel • needs assistance to travel | Code: Any mention of limited mobility, pain, weakness, or injury-related physical difficulty that prevents or restricts independent travel for follow-up care. |
| **Theme 2. Discharge process breakdown** | **Discharge Communication Limitations** | • instructions not clearly explained • difficulty remembering instructions • poor discharge communication | Code: Any mention of difficulties in how discharge information, instructions, or advice are communicated, understood, or retained by patients or caregivers. |
|  | **Limited Counselling Time** | • too little counselling time • staff too busy • rushed discharge counselling | Code: Any mention of insufficient time, workload, or competing demands limiting the amount or depth of discharge counselling provided. |
|  | **Incomplete Instruction** | • missing discharge instructions • no information on exercises • unclear medication/cast advice | Code: Any mention of essential discharge information being omitted, unclear, incomplete, or insufficiently detailed. |
|  | **Poor Comprehension** | • did not understand instructions • caregiver misunderstood advice • forgot discharge instructions | Code: Any mention of patients or caregivers not understanding, remembering, or correctly interpreting discharge instructions despite receiving an explanation. |
|  | **Caregiver Discontinuity** | • different caregiver at home • information not passed to caregiver • caregiver not present at discharge | Code: Any mention of a mismatch between the caregiver who receives discharge information and the person who subsequently provides care at home, resulting in loss or distortion of information. |
| **Theme 3. Social and community influence** | **Community Treatment Influence** | • family advice • peer influence • community advice on treatment | Code: Any mention of family members, peers, neighbours, or other community members influencing decisions about fracture treatment, recovery, or post-discharge care. |
|  | **Advice to Remove POP** | • advised to remove POP • pressure to remove plaster • community recommendation to remove cast | Code: Any mention of advice, encouragement, or pressure from others to remove plaster of Paris (POP) before the recommended time. |
|  | **Peer Recovery Narratives** | • story of another patient's recovery • comparison with friend's healing • peer experience influencing treatment | Code: Any mention of patients or caregivers using other people's fracture recovery experiences or stories to guide treatment or care decisions. |
| **Theme 4. Traditional bone setter dynamics** | **Traditional Bone Setter Utilisation** | • sought traditional bone setter • returned to bone setter after discharge • preference for traditional treatment | Code: Any mention of reasons, circumstances, or motivations for seeking traditional bone setter services after hospital discharge. |
|  | **Accessibility** | • bone setter nearby • easy access to bone setter • convenient location | Code: Any mention of the physical proximity, availability, opening hours, or ease of reaching traditional bone setter services. |
|  | **Financial Negotiability** | • negotiable payment • pay later • lower treatment cost | Code: Any mention of flexible, negotiable, deferred, or otherwise affordable payment arrangements offered by traditional bone setters. |
|  | **Relational Trust** | • trust in bone setter • familiar provider • personal attention | Code: Any mention of cultural familiarity, confidence, personal attention, previous experience, or trust that encourages use of traditional bone setter services. |
|  | **Complications After Bone Setter Care** | • swelling after bone setter care • worsening fracture condition • complication following traditional treatment | Code: Any mention of adverse outcomes, worsening symptoms, delayed recovery, or other complications following traditional bone setter intervention. |
|  | **POP Removal** | • removed POP early • cast removed by bone setter • self-removal of plaster | Code: Any mention of removing a medically applied plaster of Paris cast, including premature or non-clinically advised removal. |
|  | **Malunion** | • bone healed in wrong position • deformity after fracture • malaligned healing | Code: Any mention of improper, abnormal, or misaligned healing of a fractured bone following fracture treatment. |
| **Theme 5. Health system structural limitations** | **Workforce Constraints** | • shortage of physiotherapists • few orthopaedic staff • staff workload | Code: Any mention of insufficient numbers, availability, distribution, or workload of staff providing orthopaedic or physiotherapy services. |
|  | **Single-Provider Departments** | • one clinician covering service • single physiotherapist • department depends on one provider | Code: Any mention of a fracture, orthopaedic, or physiotherapy service relying on only one clinician or provider to deliver the service. |
|  | **Resource Constraints** | • lack of equipment • shortage of supplies • insufficient operational funds | Code: Any mention of shortages or inadequate availability of equipment, supplies, funds, space, or other operational resources needed to support follow-up care. |
|  | **Airtime Limitation** | • no airtime for follow-up calls • cannot call patients • no communication budget | Code: Any mention of inadequate funding or lack of telephone airtime to contact patients or caregivers for reminders, follow-up, or support. |
|  | **Transport Limitation** | • no outreach transport • no transport for VHT visits • outreach not facilitated | Code: Any mention of lack of transport, transport funding, or facilitation for community outreach or follow-up visits. |
|  | **Service Centralisation** | • services only at referral hospital • long distance to fracture clinic • limited services at lower facilities | Code: Any mention of fracture care or rehabilitation services being concentrated at higher-level facilities, requiring patients to travel away from their communities. |
| **Theme 6. Beliefs and misconceptions** | **Fracture Treatment Misconceptions** | • misunderstanding fracture healing • belief that hospital treatment is insufficient • incorrect treatment beliefs | Code: Any mention of inaccurate, misleading, or culturally shaped beliefs about fracture healing, treatment, rehabilitation, or hospital care. |
|  | **POP Myths** | • POP causes itching • POP causes lice • POP delays healing | Code: Any mention of beliefs or misconceptions that plaster of Paris causes problems such as lice, “rice”, itching, discomfort, or delayed healing. |
|  | **Healing Speed Misinterpretation** | • pain stopped so fracture is healed • less pain means complete healing • feels better therefore stops care | Code: Any mention of interpreting reduction or absence of pain as evidence that the fracture has healed adequately or completely. |
|  | **Spiritual Explanations** | • healing attributed to spiritual forces • ritual treatment • spiritual explanation for complications | Code: Any mention of attributing fracture healing, complications, or recovery to spiritual, ritual, supernatural, or other non-biomedical forces. |
| **Theme 7. Co-designed intervention proposals** | **Structured Discharge System** | • standard discharge process • formal discharge procedure • standardised follow-up system | Code: Any mention of a proposed standardised system for organising, documenting, and delivering fracture discharge processes. |
|  | **Discharge Checklist** | • discharge checklist • standard counselling checklist • checklist for essential discharge information | Code: Any mention of a standardised checklist or counselling framework to ensure essential discharge information is consistently provided. |
|  | **Contact Documentation** | • record caregiver phone number • document VHT contact • capture patient contact details | Code: Any mention of systematically recording patient, caregiver, telephone, address, or VHT contact information to facilitate post-discharge follow-up. |
|  | **Community Linkage Mechanism** | • facility-to-VHT referral • community follow-up linkage • formal referral to VHT | Code: Any mention of a formal process for linking discharged fracture patients with VHTs or other community-based providers. |
|  | **VHT Notification** | • notify VHT at discharge • VHT discharge alert • inform community worker | Code: Any mention of notifying or alerting a VHT when a fracture patient is discharged and requires community follow-up. |
|  | **Appointment Reminder System** | • SMS appointment reminder • follow-up phone call • review appointment reminder | Code: Any mention of using telephone calls, messages, or other communication methods to remind patients or caregivers about scheduled reviews. |
|  | **VHT Capacity Strengthening** | • VHT fracture training • VHT mentoring • community rehabilitation skills | Code: Any mention of training, mentoring, supervision, or other support intended to strengthen VHT capacity for fracture follow-up. |
|  | **POP Monitoring** | • check POP condition • monitor cast complications • community cast monitoring | Code: Any mention of VHT or community-level monitoring of plaster of Paris casts and identification of possible cast-related complications. |
|  | **Referral Triggers** | • danger signs for referral • criteria for urgent return • referral warning signs | Code: Any mention of defined danger signs, symptoms, or circumstances that should prompt referral or return to a health facility. |
|  | **Traditional Bone Setter Engagement** | • TBS referral pathway • communication with bone setters • defined TBS referral boundaries | Code: Any mention of proposed collaboration, communication, referral pathways, or defined boundaries between health services and traditional bone setters. |
|  | **Decentralised Follow-Up Services** | • follow-up at lower health centre • decentralised fracture review • community-level rehabilitation | Code: Any mention of extending basic fracture review, rehabilitation support, or follow-up closer to patients through lower-level health facilities. |
|  | **Policy and Financing Advocacy** | • district financing • Ministry policy support • staffing and resource advocacy | Code: Any mention of engaging district, Ministry of Health, or other decision-makers to secure policy support, staffing, resources, or sustainable financing for post-discharge fracture care. |
