# Supplementary material for: Co-designing a post-discharge intervention to improve mobility recovery among patients with lower limb fractures using human-centered design in greater Kampala, Uganda
Source: PLoS One. 2026 Nov 10;21(11):e0360232. doi: 10.1371/journal.pone.0360232 (PMC13750591; doi:10.1371/journal.pone.0360232)
Supplement: Supplementary file 4 [file pone.0360232.s007.docx]

### Review Report- PONE-D-26-33702

Thank you for giving me a chance to review this interesting manuscript for PLOS one.

Recommendation: **Major Revision.**

This manuscript addresses an important and clinically relevant challenge in post-discharge care for patients with lower limb fractures using a Human-Centered Design approach. The inclusion of multiple stakeholder perspectives and the focus on developing a context-specific intervention are notable strengths. However, substantial clarification is required regarding the methodological rigor and reproducibility of the HCD process, qualitative analysis, intervention development, and prototype refinement. These concerns are addressable through major revision and do not detract from the overall relevance and potential contribution of the study.

A. Introduction section

1. The Introduction describes the burden of lower limb fractures, the importance of post-discharge care, and the potential value of Human-Centered Design. However, it does not clearly distinguish how the current study advances existing knowledge beyond previous studies.
2. The manuscript introduces HCD as a participatory methodology, but does not sufficiently explain why HCD is particularly suitable for developing post-discharge fracture interventions compared with conventional intervention development approaches?
3. Although the Introduction states that research is limited, the manuscript does not explicitly identify the novel contribution of this work.
4. Language editing is recommended to reduce repetitive use of terms such as "post-discharge care" and "lower limb fractures" and to improve sentence conciseness in several paragraphs.

B. Methods:

Methods are **not sufficiently reproducible or methodologically robust in their present form.**

- Clarify the Human-Centered Design methodology, including the specific HCD framework used, any adaptations made, and the role of each stakeholder throughout all HCD phases.
- Provide explicit inclusion/exclusion criteria, participant recruitment procedures, stakeholder-specific sample composition, and explain how data saturation was achieved.
- Expand the description of qualitative data collection, including the number of FGDs, IDIs, KIIs, co-design sessions, participant composition, duration, setting, language, facilitators, and sequence of activities.
- Describe the intervention development process in greater detail, including problem definition, consensus-building, ideation, prioritization, and decision-making.
- Clarify prototype development and evaluation. Describe the prototype and its iterative refinement. If no usability or field testing was conducted, replace "prototype testing" with a more appropriate term such as "stakeholder review and refinement."
- Expand the thematic analysis methodology, including analytic approach, coding process, number of coders, codebook development, triangulation, disagreement resolution, and integration of findings across stakeholder groups.
- Clarify how the Human-Centered Design, Continuity of Care, and Proctor Implementation Outcomes frameworks were applied throughout the study.
- Strengthen reporting of research rigor, including reflexivity, interviewer characteristics, transcription/translation procedures, member checking, confidentiality, data management, and ethical procedures for participant recruitment.
- Move findings currently presented in the Methods section to the Results section.
- I would like to see Completed COREQ checklist/ interview guides/ coding framework/codebook/ intervention prototype/ intervention prioritization matrix/ HCD process diagram/ participant recruitment flow diagram. Please incorporate in supplementary material or authors can cite in methodology section.

C. Results:

Please clarify whether the participants presented in Table 2 represent patients, caregivers, or patient–caregiver dyads, provide greater transparency regarding the process used to prioritize the final intervention components, ensure that the Results section remains descriptive by relocating interpretive statements to the Discussion where appropriate, and consider supporting each major theme with quotations from multiple stakeholder groups to enhance the credibility of the findings.

D. Discussion:

Please strengthen the Discussion by explaining more explicitly how the Human-Centered Design process itself contributed to the development of the proposed intervention beyond what could have been achieved through a conventional qualitative needs assessment.

Please discuss the transferability of the findings beyond Greater Kampala, including factors that may influence implementation in other low-resource settings.

Please expand the limitations by acknowledging that the proposed intervention has not yet undergone pilot implementation or formal usability evaluation, and therefore its effectiveness remains to be established.

E. Minor comments:

Repeated phrases such as post-discharge care/lower limb fractures/healthcare providers appear repeatedly within the same paragraph. The manuscript would benefit from careful language editing to reduce repetitive use of key terms and improve sentence variety without altering scientific meaning.

Please perform a final language and copyediting review to ensure consistency in grammar and hyphenation throughout the manuscript.

The manuscript repeatedly refers to prototype testing. On other hand, the Methods describe stakeholder review and discussion rather than formal usability testing or pilot implementation. This wording is stronger than data supports. I would prefer to replace this with either stakeholder review/stakeholder validation/intervention refinement/prototype review unless actual testing occurred.

F. References:

Please undertake a comprehensive claim-to-citation audit throughout the manuscript.

Reference 35 is incorrectly attributed and should be replaced with the original COREQ publication by Tong, Sainsbury, and Craig.

Reference 43 is a systematic-review protocol and should not be cited as evidence of effectiveness.

References 2, 61, 64, and 65 require complete bibliographic information.

Please also verify the exact sources for all numerical burden estimates and replace weak or tangential references with directly relevant peer-reviewed sources where available.

Thank you
