# Supplementary material for: Co-designing a post-discharge intervention to improve mobility recovery among patients with lower limb fractures using human-centered design in greater Kampala, Uganda
Source: PLoS One. 2026 Nov 10;21(11):e0360232. doi: 10.1371/journal.pone.0360232 (PMC13750591; doi:10.1371/journal.pone.0360232)
Supplement: Supplementary file 5 [file pone.0360232.s008.docx]

**18^th^, September 2026**

**MANUSCRIPT TITTLE; CO-DESIGNING A POST-DISCHARGE INTERVENTION TO IMPROVE MOBILITY RECOVERY AMONG PATIENTS WITH LOWER LIMB FRACTURES USING HUMAN-CENTERED DESIGN IN GREATER KAMPALA, UGANDA”** (Manuscript ID: **PONE-D-26-33702**)

Dear Reviewers,

Thank you so much for taking the time to review our manuscript. We sincerely appreciate your constructive and insightful comments. Your feedback has helped us to improve the clarity, methodological rigor, and overall quality of the manuscript.

Kindly find below our point-by-point responses to the comments. We have carefully considered each comment and made the necessary revisions to the manuscript. Where appropriate, we have provided explanations and indicated the corresponding changes made in the revised manuscript highlighted in blue print.

We thank you again for your valuable time, expertise, and constructive feedback.

Yours sincerely,

**Swaibu Zziwa**
Corresponding Author

| **Reviewer’s Comment** | **Authors’ Response** |
| --- | --- |
| **Reviewer #1** | |
| Given the growing burden of lower limb fractures and the critical importance of post-discharge | Thank you for this important comment. We have now revised this section to provide evidence on the burden of bone fractures in Uganda and to emphasize the significant importance of post-discharge care in supporting recovery among patients with lower-limb fractures. (Page 4, line 84-89) and page 5, line 97-102) |
| **Reviewer #2:** |  |
| **A. Introduction section** 1. The Introduction describes the burden of lower limb fractures, the importance of post-discharge care, and the potential value of Human-Centered Design. However, it does not clearly distinguish how the current study advances existing knowledge beyond previous studies. | Thank you for this important comment. We have revised the Introduction to clearly highlight the knowledge gap and distinguish our study from previous research. (Page 6 & 7, line 132-139) |
| 2. The manuscript introduces HCD as a participatory methodology, but does not sufficiently explain why HCD is particularly suitable for developing post-discharge fracture interventions compared with conventional intervention development approaches? | Thank you for this important comment. We have now revised the manuscript to provide a clearer justification for the suitability of Human-Centered Design (HCD) compared with conventional intervention-development approaches. (Page 6, line 116-124) and (line 125-131) |
| 3. Although the Introduction states that research is limited, the manuscript does not explicitly identify the novel contribution of this work. | Thank you for this important comment. We have now revised the Introduction to explicitly highlight the novel contribution of our study. Previous studies in Uganda have primarily focused on the burden and clinical management of fractures. To the best of Author’s knowledge, no study has used an HCD approach to co-design a post-discharge intervention to improve mobility recovery among patients with lower limb fractures specifically in Uganda. (Page 6 & 7, line 132-139) |
| 4. Language editing is recommended to reduce repetitive use of terms such as "post-discharge care" and "lower limb fractures" and to improve sentence conciseness in several paragraphs. | Thank you for this comment. We have now carefully revised the manuscript for **language, clarity, and conciseness**. We reduced unnecessary repetition of terms “post-discharge care” and “lower limb fractures”. In addition, the entire manuscript was **copy-edited by an independent language expert** to improve grammar, sentence structure, readability, and overall clarity. |
| **B. Methods:** |  |
| Methods are not sufficiently reproducible or methodologically robust in their present form. - Clarify the Human-Centered Design methodology, including the specific HCD framework used, any adaptations made, and the role of each stakeholder throughout all HCD phases. | Thank you for this important comment. The HCD framework used in the present study was a **participatory and iterative design approach,** informed by the **experiences, needs, challenges, preferences, and recommendations generated directly by study participants.** These findings formed the basis for identifying priority problems and co-designing the proposed post-discharge intervention. (Page 7, line 143-145)  We have also revised the method section to clearly describe the contributions of each stakeholder during the HCD intervention development process. (Page 12, line 265-268) |
| - Provide explicit inclusion/exclusion criteria, participant recruitment procedures, stakeholder-specific sample composition, and explain how data saturation was achieved. | Thank you for this important comment. We have now revised the method section to provide **more detail on participant eligibility, recruitment, stakeholder composition and sampling procedure (Page 8, line 167-172 and page 8 & 9, line 178-183).**  Data collection continued until **data saturation was reached (Page 9&10, line 200-203), page 10, line 212-216)** |
| - Expand the description of qualitative data collection, including the number of FGDs, IDIs, KIIs, co-design sessions, participant composition, duration, setting, language, facilitators, and sequence of activities. | Thank you for this important comment. We have expanded the Methods section to provide a more detailed description of the **qualitative data collection and co-design process.** The revised manuscript now specifies the **number and type of qualitative sessions conducted (FGDs, IDIs, and KIIs), participant composition, the stakeholder groups represented, the duration and setting of the sessions, the language used, and the roles of the facilitators and research team (**Page 9-12, line 184-255**).** |
| - Describe the intervention development process in greater detail, including problem definition, consensus-building, ideation, prioritization, and decision-making. | Thank you for this important comment. We have now expanded the description of the intervention development process in the revised manuscript to provide greater detail on how the HCD process informed problem definition, consensus-building, ideation, prioritization, and decision-making. (Page 12-14, line 256-299) |
| - Clarify prototype development and evaluation. Describe the prototype and its iterative refinement. If no usability or field testing was conducted, replace "prototype testing" with a more appropriate term such as "stakeholder review and refinement." | Thank you for this important comment. We have revised this section to clarify that the intervention prototype was **developed from the ideas generated during the ideation phase and subsequently presented to participants for stakeholder review and iterative refinement.**  Since in the present study we did **not conduct prototype testing or field testing**, we have replaced the term **“prototype testing”** with **“stakeholder review and refinement” as** throughout the manuscript as guided. (Page 13-14, line 284-299) |
| - Expand the thematic analysis methodology, including analytic approach, coding process, number of coders, codebook development, triangulation, disagreement resolution, and integration of findings across stakeholder groups. | Thank you for this important comment. We have now expanded the thematic analysis section to clarify the **analytic approach, coding process, number of coders, codebook development, disagreement resolution, and triangulation.** (Page 15-17, line 329-352) |
| - Clarify how the Human-Centered Design, Continuity of Care, and Proctor Implementation Outcomes frameworks were applied throughout the study. | Thank you for this important comment. We have revised the manuscript to clarify how the three frameworks were applied during the intervention development process. Specifically, ***HCD guided* the overall participatory and iterative co-design process**, the ***Continuity of Care framework* was used to identify gaps and intervention opportunities across the post-discharge care pathway,** and the ***Proctor Implementation Outcomes framework* guided the prioritization and refinement of intervention strategies based on their implementation potential.** (14-15, line 300-328) |
| - Strengthen reporting of research rigor, including reflexivity, interviewer characteristics, transcription/translation procedures, member checking, confidentiality, data management, and ethical procedures for participant recruitment. | Thank you for this important comment. We have strengthened the reporting of qualitative research rigor in the revised manuscript by providing additional details on the **characteristics and roles of the interviewers, reflexivity, transcription and translation procedures** (Page 11, line 232-242)**, confidentiality and data management, and procedures for participant recruitment and informed consent (page 17-18, line 373-388).**We have also clarified the measures undertaken to enhance the **credibility, dependability, confirmability, and transferability** of the findings (Page 16-17, line 353-368) |
| - Move findings currently presented in the Methods section to the Results section | Thank you for this helpful comment. We have now revised the manuscript to **separate the Results from the** Method **section** |
| - I would like to see Completed COREQ checklist/ interview guides/ coding framework/codebook/ intervention prototype/ intervention prioritization matrix/ HCD process diagram/ participant recruitment flow diagram. Please incorporate in supplementary material or authors can cite in methodology section. | Thank you for this important comment. We have now attached the **completed COREQ checklist, interview guides, coding framework/codebook, intervention prototype, intervention prioritization matrix, HCD process diagram, and participant recruitment flow diagram** as supplementary files. We have also **cited the supplementary files in the methods section** |
| **C. Results** |  |
| Please clarify whether the participants presented in Table 2 represent patients, caregivers, or patient–caregiver dyads, provide greater transparency regarding the process used to prioritize the final intervention components, ensure that the Results section remains descriptive by relocating interpretive statements to the Discussion where appropriate, and consider supporting each major theme with quotations from multiple stakeholder groups to enhance the credibility of the findings | Thank you for this helpful comment. We have now clarified in the revised manuscript that the participants presented in **Table 2 comprised 15 patients and 15 caregivers**, who were considered as separate participant groups and **not as patient–caregiver dyads.**  We have also provided greater transparency regarding the process used to **prioritize the final intervention components.** The prioritization process and resulting intervention components are presented in the **Participant-Informed Intervention Prioritization Matrix (Supplementary File 4, Page 15, line 319),** which is now cited in the Methods section.  We have revised the **Results section** to ensure that it remains primarily descriptive, with interpretive statements and explanations of the broader implications of the findings relocated to the **Discussion section** where appropriate.  We have also strengthened the presentation of the qualitative findings by incorporating **supporting quotations from multiple stakeholder groups,** including healthcare providers, community health workers, patients, and caregivers, where relevant. |
| **D. Discussion:** |  |
| Please strengthen the Discussion by explaining more explicitly how the Human-Centered Design process itself contributed to the development of the proposed intervention beyond what could have been achieved through a conventional qualitative needs assessment. Please discuss the transferability of the findings beyond Greater Kampala, including factors that may influence implementation in other low-resource settings. Please expand the limitations by acknowledging that the proposed intervention has not yet undergone pilot implementation or formal usability evaluation, and therefore its effectiveness remains to be established. | Thank you for this important comment. We have now revised the Discussion to clarify the contribution of the HCD process beyond conventional qualitative needs assessment. In particular, HCD enabled stakeholders to share their experiences, identify barriers and needs, and to develop contextually appropriate solutions through a **participatory and iterative process,** which could not readily be achieved through conventional qualitative needs assessment (Page 39, line 726-741)  We have also expanded the Discussion to address the **transferability of the findings beyond Greater Kampala (Page 43, 810-822)**  We have revised the limitation section to acknowledge that the proposed intervention has **not yet undergone pilot testing or formal usability evaluation,** and therefore its feasibility, acceptability, and effectiveness remain to be established (Page 44- line 846-848) |
| **F. References:** Please undertake a comprehensive claim-to-citation audit throughout the manuscript. Reference 35 is incorrectly attributed and should be replaced with the original COREQ publication by Tong, Sainsbury, and Craig.  Reference 43 is a systematic-review protocol and should not be cited as evidence of effectiveness.  References 2, 61, 64, and 65 require complete bibliographic information. | Thank you for this important comment. We have undertaken a comprehensive claim-to-citation audit throughout the manuscript to ensure that all claims are appropriately supported by relevant and accurate references.  We have also corrected Reference 35 and replaced the incorrectly attributed citation with the **original COREQ publication by Tong, Sainsbury, and Craig (2007) -Reference 41.**  Thank you for this important observation. We have removed Reference 43 as evidence of effectiveness community-based rehabilitation. We have replaced it with an appropriate published study reporting evidence on the effectiveness of community-based rehabilitation -Reference 49.  Thank you for this comment. We have reviewed References 2, 61, 64, and 65 and completed the missing bibliographic information,  Reference 61- it’s now 66, 64 its 68, and 65, replaced with 67 in the revised manuscript |
