# Supplementary material for: Co-designing a post-discharge intervention to improve mobility recovery among patients with lower limb fractures using human-centered design in greater Kampala, Uganda
Source: PLoS One. 2026 Nov 10;21(11):e0360232. doi: 10.1371/journal.pone.0360232 (PMC13750591; doi:10.1371/journal.pone.0360232)
Supplement: Peer review history

# Peer review: Decision Letter 0

**DOI:** 10.1371/journal.pone.0360232.r001

**Authors:** Xiaoen Wei

14 Aug 2026

PONE-D-26-33702CO-DESIGNING A POST-DISCHARGE INTERVENTION TO IMPROVE MOBILITY RECOVERY AMONG PATIENTS WITH LOWER LIMB FRACTURES USING HUMAN-CENTERED DESIGN (HCD) IN GREATER KAMPALA, UGANDAPLOS One

Dear Dr. Zziwa,

Thank you for submitting your manuscript to PLOS ONE. After careful consideration, we feel that it has merit but does not fully meet PLOS ONE’s publication criteria as it currently stands. Therefore, we invite you to submit a revised version of the manuscript that addresses the points raised during the review process.

Please submit your revised manuscript by Oct 13 2026 11:59PM. If you will need more time than this to complete your revisions, please reply to this message or contact the journal office at plosone@plos.org. When you're ready to submit your revision, log on to https://www.editorialmanager.com/pone/ and select the 'Submissions Needing Revision' folder to locate your manuscript file.

Please include the following items when submitting your revised manuscript:

- A letter that responds to each point raised by the academic editor and reviewer(s). You should upload this letter as a separate file labeled 'Response to Reviewers'.
- A marked-up copy of your manuscript that highlights changes made to the original version. You should upload this as a separate file labeled 'Revised Manuscript with Track Changes'.
- An unmarked version of your revised paper without tracked changes. You should upload this as a separate file labeled 'Manuscript'.

If you would like to make changes to your financial disclosure, please include your updated statement in your cover letter. Guidelines for resubmitting your figure files are available below the reviewer comments at the end of this letter.

If applicable, we recommend that you deposit your laboratory protocols in protocols.io to enhance the reproducibility of your results. Protocols.io assigns your protocol its own identifier (DOI) so that it can be cited independently in the future. For instructions see: https://journals.plos.org/plosone/s/submission-guidelines#loc-laboratory-protocols. Additionally, PLOS ONE offers an option for publishing peer-reviewed Lab Protocol articles, which describe protocols hosted on protocols.io. Read more information on sharing protocols at https://plos.org/protocols?utm_medium=editorial-email&utm_source=authorletters&utm_campaign=protocols.

As the corresponding author, your ORCID iD is verified in the submission system and will appear in the published article. PLOS supports the use of ORCID, and we encourage all coauthors to register for an ORCID iD and use it as well. Please encourage your coauthors to verify their ORCID iD within the submission system before final acceptance, as unverified ORCID iDs will not appear in the published article. *Only*  the individual author can complete the verification step; PLOS staff *cannot*  verify ORCID iDs on behalf of authors.

We look forward to receiving your revised manuscript.

Kind regards,

Xiaoen Wei

Academic Editor

PLOS One

Journal Requirements:

When submitting your revision, we need you to address these additional requirements.

1. Please ensure that your manuscript meets PLOS ONE's style requirements, including those for file naming. The PLOS ONE style templates can be found at

https://journals.plos.org/plosone/s/file?id=wjVg/PLOSOne_formatting_sample_main_body.pdf and

https://journals.plos.org/plosone/s/file?id=ba62/PLOSOne_formatting_sample_title_authors_affiliations.pdf

2. We note that the grant information you provided in the ‘Funding Information’ and ‘Financial Disclosure’ sections do not match.

When you resubmit, please ensure that you provide the correct grant numbers for the awards you received for your study in the ‘Funding Information’ section.

3. We note that you have indicated that there are restrictions to data sharing for this study. For studies involving human research participant data or other sensitive data, we encourage authors to share de-identified or anonymized data. However, when data cannot be publicly shared for ethical reasons, we allow authors to make their data sets available upon request. For information on unacceptable data access restrictions, please see http://journals.plos.org/plosone/s/data-availability#loc-unacceptable-data-access-restrictions.

Before we proceed with your manuscript, please address the following prompts:

a) If there are ethical or legal restrictions on sharing a de-identified data set, please explain them in detail (e.g., data contain potentially identifying or sensitive patient information, data are owned by a third-party organization, etc.) and who has imposed them (e.g., a Research Ethics Committee or Institutional Review Board, etc.). Please also provide contact information for a data access committee, ethics committee, or other institutional body to which data requests may be sent.

b) If there are no restrictions, please upload the minimal anonymized data set necessary to replicate your study findings to a stable, public repository and provide us with the relevant URLs, DOIs, or accession numbers. Please see http://www.bmj.com/content/340/bmj.c181.long for guidelines on how to de-identify and prepare clinical data for publication. For a list of recommended repositories, please see https://journals.plos.org/plosone/s/recommended-repositories. You also have the option of uploading the data as Supporting Information files, but we would recommend depositing data directly to a data repository if possible.

Please update your Data Availability statement in the submission form accordingly.

4. Please include captions for your Supporting Information files at the end of your manuscript, and update any in-text citations to match accordingly. Please see our Supporting Information guidelines for more information: http://journals.plos.org/plosone/s/supporting-information.

5. If the reviewer comments include a recommendation to cite specific previously published works, please review and evaluate these publications to determine whether they are relevant and should be cited. There is no requirement to cite these works unless the editor has indicated otherwise.

[Note: HTML markup is below. Please do not edit.]

Reviewers' comments:

Reviewer's Responses to Questions

**Comments to the Author**

1. Is the manuscript technically sound, and do the data support the conclusions?

The manuscript must describe a technically sound piece of scientific research with data that supports the conclusions. Experiments must have been conducted rigorously, with appropriate controls, replication, and sample sizes. The conclusions must be drawn appropriately based on the data presented.

Reviewer #1: Yes

Reviewer #2: Yes

**********

2. Has the statistical analysis been performed appropriately and rigorously?

Reviewer #1: Yes

Reviewer #2: Yes

**********

3. Have the authors made all data underlying the findings in their manuscript fully available?

The PLOS Data policy requires authors to make all data underlying the findings described in their manuscript fully available without restriction, with rare exception (please refer to the Data Availability Statement in the manuscript PDF file). The data should be provided as part of the manuscript or its supporting information, or deposited to a public repository. For example, in addition to summary statistics, the data points behind means, medians and variance measures should be available. If there are restrictions on publicly sharing data—e.g. participant privacy or use of data from a third party—those must be specified.

Reviewer #1: Yes

Reviewer #2: No

**********

4. Is the manuscript presented in an intelligible fashion and written in standard English?

PLOS ONE does not copyedit accepted manuscripts, so the language in submitted articles must be clear, correct, and unambiguous. Any typographical or grammatical errors should be corrected at revision, so please note any specific errors here.

Reviewer #1: Yes

Reviewer #2: Yes

**********

5. Review Comments to the Author

Please use the space provided to explain your answers to the questions above. You may also include additional comments for the author, including concerns about dual publication, research ethics, or publication ethics. (Please upload your review as an attachment if it exceeds 20,000 characters)

Reviewer #1: Given the growing burden of lower limb fractures and the critical importance of post-discharge

101 care, there is a need for context-specific interventions that strengthen the transition from the

102 hospital to the community. Therefore, this study aimed to co-design a post-discharge intervention

6

103 to improve recovery among patients with lower limb fractures in Greater Kampala using a Human 104 Centered Design approach.

Reviewer #2: Thank you for giving me a chance to review this interesting manuscript for PLOS one.

Recommendation: Major Revision.

This manuscript addresses an important and clinically relevant challenge in post-discharge care for patients with lower limb fractures using a Human-Centered Design approach. The inclusion of multiple stakeholder perspectives and the focus on developing a context-specific intervention are notable strengths. However, substantial clarification is required regarding the methodological rigor and reproducibility of the HCD process, qualitative analysis, intervention development, and prototype refinement. These concerns are addressable through major revision and do not detract from the overall relevance and potential contribution of the study.

A. Introduction section

1. The Introduction describes the burden of lower limb fractures, the importance of post-discharge care, and the potential value of Human-Centered Design. However, it does not clearly distinguish how the current study advances existing knowledge beyond previous studies.

2. The manuscript introduces HCD as a participatory methodology, but does not sufficiently explain why HCD is particularly suitable for developing post-discharge fracture interventions compared with conventional intervention development approaches?

3. Although the Introduction states that research is limited, the manuscript does not explicitly identify the novel contribution of this work.

4. Language editing is recommended to reduce repetitive use of terms such as "post-discharge care" and "lower limb fractures" and to improve sentence conciseness in several paragraphs.

B. Methods:

Methods are not sufficiently reproducible or methodologically robust in their present form.

- Clarify the Human-Centered Design methodology, including the specific HCD framework used, any adaptations made, and the role of each stakeholder throughout all HCD phases.

- Provide explicit inclusion/exclusion criteria, participant recruitment procedures, stakeholder-specific sample composition, and explain how data saturation was achieved.

- Expand the description of qualitative data collection, including the number of FGDs, IDIs, KIIs, co-design sessions, participant composition, duration, setting, language, facilitators, and sequence of activities.

- Describe the intervention development process in greater detail, including problem definition, consensus-building, ideation, prioritization, and decision-making.

- Clarify prototype development and evaluation. Describe the prototype and its iterative refinement. If no usability or field testing was conducted, replace "prototype testing" with a more appropriate term such as "stakeholder review and refinement."

- Expand the thematic analysis methodology, including analytic approach, coding process, number of coders, codebook development, triangulation, disagreement resolution, and integration of findings across stakeholder groups.

- Clarify how the Human-Centered Design, Continuity of Care, and Proctor Implementation Outcomes frameworks were applied throughout the study.

- Strengthen reporting of research rigor, including reflexivity, interviewer characteristics, transcription/translation procedures, member checking, confidentiality, data management, and ethical procedures for participant recruitment.

- Move findings currently presented in the Methods section to the Results section.

- I would like to see Completed COREQ checklist/ interview guides/ coding framework/codebook/ intervention prototype/ intervention prioritization matrix/ HCD process diagram/ participant recruitment flow diagram. Please incorporate in supplementary material or authors can cite in methodology section.

C. Results:

Please clarify whether the participants presented in Table 2 represent patients, caregivers, or patient–caregiver dyads, provide greater transparency regarding the process used to prioritize the final intervention components, ensure that the Results section remains descriptive by relocating interpretive statements to the Discussion where appropriate, and consider supporting each major theme with quotations from multiple stakeholder groups to enhance the credibility of the findings.

D. Discussion:

Please strengthen the Discussion by explaining more explicitly how the Human-Centered Design process itself contributed to the development of the proposed intervention beyond what could have been achieved through a conventional qualitative needs assessment.

Please discuss the transferability of the findings beyond Greater Kampala, including factors that may influence implementation in other low-resource settings.

Please expand the limitations by acknowledging that the proposed intervention has not yet undergone pilot implementation or formal usability evaluation, and therefore its effectiveness remains to be established.

E. Minor comments:

Repeated phrases such as post-discharge care/lower limb fractures/healthcare providers appear repeatedly within the same paragraph. The manuscript would benefit from careful language editing to reduce repetitive use of key terms and improve sentence variety without altering scientific meaning.

Please perform a final language and copyediting review to ensure consistency in grammar and hyphenation throughout the manuscript.

The manuscript repeatedly refers to prototype testing. On other hand, the Methods describe stakeholder review and discussion rather than formal usability testing or pilot implementation. This wording is stronger than data supports. I would prefer to replace this with either stakeholder review/stakeholder validation/intervention refinement/prototype review unless actual testing occurred.

F. References:

Please undertake a comprehensive claim-to-citation audit throughout the manuscript.

Reference 35 is incorrectly attributed and should be replaced with the original COREQ publication by Tong, Sainsbury, and Craig.

Reference 43 is a systematic-review protocol and should not be cited as evidence of effectiveness.

References 2, 61, 64, and 65 require complete bibliographic information.

Please also verify the exact sources for all numerical burden estimates and replace weak or tangential references with directly relevant peer-reviewed sources where available.

Thank you

**********

6. PLOS authors have the option to publish the peer review history of their article (what does this mean?). If published, this will include your full peer review and any attached files.

If you choose “no”, your identity will remain anonymous but your review may still be made public.

**Do you want your identity to be public for this peer review?**  For information about this choice, including consent withdrawal, please see our Privacy Policy.

Reviewer #1: No

Reviewer #2: **Yes:**  Faizan Iqbal

**********

[NOTE: If reviewer comments were submitted as an attachment file, they will be attached to this email and accessible via the submission site. Please log into your account, locate the manuscript record, and check for the action link "View Attachments". If this link does not appear, there are no attachment files.]

To ensure your figures meet our technical requirements, please review our figure guidelines: https://journals.plos.org/plosone/s/figures

You may also use PLOS’s free figure tool, NAAS, to help you prepare publication quality figures: https://journals.plos.org/plosone/s/figures#loc-tools-for-figure-preparation.

NAAS will assess whether your figures meet our technical requirements by comparing each figure against our figure specifications.

Attachment Submitted filename: Review_PLOS.docx

# Author comment: Author response to Decision Letter 1

**DOI:** 10.1371/journal.pone.0360232.r002

21 Sep 2026

We thank the Academic Editor and reviewers for their careful review of our manuscript and for their constructive comments and suggestions. We have addressed all the comments raised and revised the manuscript accordingly.

A detailed point-by-point response to each comment has been provided in the uploaded Response to Reviewers document. All corresponding changes have been incorporated into the revised manuscript and are highlighted in blue in the marked-up version for ease of reference.

We appreciate the valuable feedback, which has helped us improve the clarity, methodological reporting, and overall quality of the manuscript.

Attachment Submitted filename: Response to Reviewers.docx.docx

# Editor report: Decision Letter 1

**DOI:** 10.1371/journal.pone.0360232.r003

**Authors:** Xiaoen Wei

25 Sep 2026

CO-DESIGNING A POST-DISCHARGE INTERVENTION TO IMPROVE MOBILITY RECOVERY AMONG PATIENTS WITH LOWER LIMB FRACTURES USING HUMAN-CENTERED DESIGN IN GREATER KAMPALA, UGANDA

PONE-D-26-33702R1

Dear Dr. Zziwa,

We’re pleased to inform you that your manuscript has been judged scientifically suitable for publication and will be formally accepted for publication once it meets all outstanding technical requirements.

Within one week, you’ll receive an e-mail detailing the required amendments. When these have been addressed, you’ll receive a formal acceptance letter and your manuscript will be scheduled for publication.

An invoice will be generated when your article is formally accepted. Please note, if your institution has a publishing partnership with PLOS and your article meets the relevant criteria, all or part of your publication costs will be covered. Please make sure your user information is up-to-date by logging into Editorial Manager at Editorial Manager® and clicking the ‘Update My Information' link at the top of the page. For questions related to billing, please contact billing support.

If your institution or institutions have a press office, please notify them about your upcoming paper to help maximize its impact. If they’ll be preparing press materials, please inform our press team as soon as possible -- no later than 48 hours after receiving the formal acceptance. Your manuscript will remain under strict press embargo until 2 pm Eastern Time on the date of publication. For more information, please contact onepress@plos.org.

Kind regards,

Xiaoen Wei

Academic Editor

PLOS One

Additional Editor Comments (optional):

Reviewers' comments:

# Editor report: Acceptance letter

**DOI:** 10.1371/journal.pone.0360232.r004

**Authors:** Xiaoen Wei

PONE-D-26-33702R1

PLOS One

Dear Dr. Zziwa,

I'm pleased to inform you that your manuscript has been deemed suitable for publication in PLOS One. Congratulations! Your manuscript is now being handed over to our production team.

At this stage, our production department will prepare your paper for publication. This includes ensuring the following:

* All references, tables, and figures are properly cited

* All relevant supporting information is included in the manuscript submission,

* There are no issues that prevent the paper from being properly typeset

You will receive further instructions from the production team, including instructions on how to review your proof when it is ready. Please keep in mind that we are working through a large volume of accepted articles, so please give us a few days to review your paper and let you know the next and final steps.

Lastly, if your institution or institutions have a press office, please let them know about your upcoming paper now to help maximize its impact. If they'll be preparing press materials, please inform our press team within the next 48 hours. Your manuscript will remain under strict press embargo until 2 pm Eastern Time on the date of publication. For more information, please contact onepress@plos.org.

You will receive an invoice from PLOS for your publication fee after your manuscript has reached the completed accept phase. If you receive an email requesting payment before acceptance or for any other service, this may be a phishing scheme. Learn how to identify phishing emails and protect your accounts at https://explore.plos.org/phishing.

If we can help with anything else, please email us at customercare@plos.org.

Thank you for submitting your work to PLOS One and supporting open access.

Kind regards,

PLOS One Editorial Office Staff

on behalf of

Dr. Xiaoen Wei

Academic Editor

PLOS One
